# CTPS1 is an unexplored vulnerability in breast and ovarian cancer

**DOI:** 10.7150/thno.134720

**Published:** 2026-07-20

**Authors:** Xiyin Wang, Michael J. Emch, Lauren A. Voll, Rebecca Epp, Esther P. B. Rodman, Noa J. Odell, Hannah M. Smith, Nicole A. Pearson, Xiaonan Hou, Ya Li, Melissa C. Larson, Ann L. Oberg, Taro Hitosugi, Matthew P. Goetz, Scott H. Kaufmann, S. John Weroha, Philip A. Beer, John R. Hawse

**Affiliations:** 1 Graduate School of Biomedical Sciences, Mayo Clinic, Rochester, MN, USA.; 2 Department of Biochemistry and Molecular Biology, Mayo Clinic, Rochester, MN, USA.; 3 Department of Laboratory Medicine and Pathology, Mayo Clinic, Rochester, MN, USA.; 4 Case Western Reserve University, Cleveland, OH, USA.; 5 Vincent Center for Reproductive Biology, Massachusetts General Hospital, Boston, MA, USA.; 6 Department of Oncology, Mayo Clinic, Rochester, MN, USA.; 7 Department of Quantitative Health Sciences, Mayo Clinic, Rochester, MN, USA.; 8 Step Pharma, Saint-Genis-Pouilly, France.

**Keywords:** triple negative breast cancer, ovarian cancer, drug resistance, CTPS1, targeted therapy

## Abstract

Triple negative breast cancer (TNBC) and ovarian cancer share many molecular features and are primarily treated with surgical resection and aggressive chemotherapy regimens. Unfortunately, survival rates for patients with advanced metastatic disease are poor, highlighting the need for innovative therapeutic approaches.

**Methods:**

Using the DepMap database, we first sought to identify genes that were highly expressed and more essential for proliferation/viability in TNBC cells relative to other breast cancer subtypes. Candidate genes were validated using gene-specific siRNAs in a panel of TNBC and estrogen receptor positive breast cancer cells. CTPS1 expression, and its functional significance, was further evaluated in ovarian cancer models, including chemotherapy- and PARP inhibitor-resistant cell lines. Pharmacologic inhibition was assessed using STP938, a first-in-class selective CTPS1 inhibitor, in TNBC and ovarian cancer cells as well as in *ex vivo* and *in vivo* patient-derived xenografts (PDX).

**Results:**

Six genes (CTPS1, HUS1, PRKRA, RAD1, RAD9A, and RHOA) were identified as potential TNBC selective dependencies. Among these, CTPS1 was prioritized for further study given that it was highly expressed, further upregulated in chemotherapy- and PARP inhibitor-resistant cell lines, and resulted in the greatest anti-neoplastic effects when depleted. Knockdown of CTPS1 confirmed its selective essentiality and resulted in rapid and durable S-phase cell cycle arrest. Pharmacologic inhibition of CTPS1 with STP938 led to robust anti-neoplastic effects at nM concentrations across both chemotherapy-sensitive and -resistant TNBC and ovarian cancer cell lines. Significant anti-neoplastic activity was observed in 6 independent *ex vivo* ovarian cancer PDX models. Further, STP938 significantly inhibited progression of an ovarian cancer PDX model *in vivo*.

**Conclusion:**

These findings identify CTPS1 as a critical dependency in TNBC and ovarian cancer. Selective pharmacologic inhibition of CTPS1 using STP938 is a potent inhibitor of tumor cell proliferation/viability and has anti-cancer activity in patient derived *ex vivo* and *in vivo* tumor models. These findings suggest that therapeutic targeting of CTPS1 represents an alternative approach for the management of patients with advanced and aggressive forms of these diseases.

## Introduction

Breast cancer accounts for nearly a quarter of all cancers in women with an estimated 2.3 million new diagnoses 1, 2 and over 670,000 deaths annually 3, 4. Triple-negative breast cancer (TNBC) is an aggressive subtype characterized by lack of estrogen receptor alpha (ERα) and progesterone receptor (PR) expression in the absence of HER2 amplification, and accounts for ~15% of all breast cancer diagnoses 5-7. Systemic chemotherapy is the backbone of treatment for TNBC using a combination of anthracyclines and taxanes in the neoadjuvant or adjuvant setting 8-10. This approach is effective, and likely curative, for a subset of patients 11, 12. However, for the approximately 40% of patients that experience a recurrence within 5 years of diagnosis 13-15, subsequent treatment with a variety of agents offers modest benefit at best, and essentially all recurrent TNBC patients will succumb to their disease 12.

Like TNBC, ovarian cancer is one of the most highly aggressive and lethal gynecologic malignancies, being the eighth most common cause of cancer related death in women worldwide 16, 17. High grade serous ovarian cancer (HGSOC) is the most common subtype, and despite standard-of-care debulking surgery and neoadjuvant and/or adjuvant chemotherapy (primarily carboplatin and paclitaxel), median disease-free survival (DFS) is dismal (1.7 years) 18. Recurrent disease is typically less responsive to subsequent therapy, and the recent uptake of targeted therapies such as PARP inhibitors (poly (ADP-ribose) polymerase inhibitor, PARPi) and anti-angiogenic agents have done little to improve overall survival 19, 20. These realities underscore the urgent need to identify alternative therapeutic approaches that are informed by biomarker-driven clinical trials, for the treatment of advanced and therapy resistant forms of HGSOC.

Highly proliferative cells, such as cancer cells, are reliant on several cellular processes, including DNA replication, RNA expression, and phospholipid synthesis, for support of cell division 21. Central to these processes is nucleotide metabolism, and as such, many of the existing and broadly applicable anticancer drugs are analogs of nucleotide metabolites (cytarabine, 5-fluorouracil, and gemcitabine) 22. While effective for cancer cell killing, these drugs indiscriminately impact all highly proliferative cells, leading to substantial, and in some cases intolerable, side effects. Thus, there is significant interest in developing alternative therapeutic approaches to selectively disrupt nucleotide metabolism in cancer cells. The high demand for nucleotide synthesis renders malignant cells dependent on multiple enzymes such as CTP synthase 1 (CTPS1), as has been demonstrated in lymphoma 23. CTPS1 is one of two enzymes (the other being CTPS2) responsible for the conversion of uridine triphosphate (UTP) to cytidine triphosphate (CTP) 24 and is the rate-limiting step in the de novo synthesis of CTP 25, 26. Emerging evidence suggests that dysregulation of nucleotide metabolism can drive tumor growth and induce therapeutic resistance 27-29, raising the possibility that selective inhibition of CTPS1 is a vulnerability in advanced neoplasms.

Here, we describe the utilization of existing DepMap CRISPR screen data whereby we identified 6 genes whose expression was more essential for proliferation/viability of TNBC cells relative to other subtypes of breast cancer cells. Through exploratory studies we demonstrated that one of these genes, CTPS1, is overexpressed in most TNBC and ovarian cancer cell lines compared to representative benign controls. Interestingly, CTPS1 expression was further elevated in chemotherapy- and PARPi-resistant models. The importance of CTPS1 was genomically validated using gene-specific siRNAs in a panel of TNBC and ovarian cancer cells. Using the first-in-class and orally bioavailable CTPS1 inhibitor, STP938, robust anti-neoplastic activity was demonstrated across multiple *in vitro*, *ex vivo*, and *in vivo* models. These findings indicate that many highly aggressive and advanced forms of TNBC and ovarian cancer are reliant on CTPS1, and as such, selective inhibition of CTPS1 represents an alternative approach for the treatment of these malignancies.

## Materials and Methods

### Bioinformatic analyses

Gene dependency data were obtained from the DepMap portal (https://depmap.org/portal) (version 23Q2) 30, focusing on genome-wide CRISPR knockout datasets derived from breast cancer cell lines. Breast cancer cell lines were stratified according to receptor subtype annotations provided within DepMap. For every gene, median essentiality scores were calculated for all TNBC cell lines and compared to median gene essentiality scores of ERα^+^ breast cancer cells. Differential essentiality scores were generated and ranked with negative gene effect scores indicating a higher reliance on a given gene for proliferation/survival in TNBC models. Statistically significant differences were determined using pairwise comparison ANOVAs with post hoc corrections. Candidate genes were prioritized based on statistical significance (P < 0.05). GraphPad Prism software (Version 10.6.1) was used to generate forest plots summarizing associations of gene expression with overall survival and clinicopathologic features of breast tumors using data from the Kaplan-Meier plotter database (http://kmplot.com) 31. Gene expression analyses were performed using the TNMplot database (https://tnmplot.com) 32 to compare mRNA expression levels between normal, tumor, and metastatic tissues. The MammOnc-DB database 33 was used to analyze RNA and protein expression levels of select genes, including comparisons of normal versus tumor samples and subtype-specific expression profiling. Publicly available transcriptomic and proteomic datasets were additionally analyzed to evaluate gene expression patterns 34-36.

### Cell culture

Breast cancer, ovarian cancer, and non-malignant control (MCF10A and FT282) cell lines were obtained from ATCC or collaborators and maintained under recommended culture conditions in DMEM/F12 (JangoCell, Fitchburg, WI) or RPMI 1640 (Gibco, Thermo Fisher Scientific, Waltham, MA) medium supplemented with 10% FBS (Gemini Bioproducts, Sacramento, CA) and 1% antibiotic-antimycotic (Gibco, Thermo Fisher Scientific, Waltham, MA). Cells were grown at 37 °C in a humidified incubator with 5% CO_2_. Chemotherapy-resistant or PARPi-resistant cells were generated through chronic drug exposure over several months and validated with increased IC50 values relative to parental controls as previously described 37-42. Briefly, doxorubicin-resistant (D-R) and paclitaxel-resistant (P-R) MDA-MB-231 and MDA-MB-468 TNBC models were generated using repeated 2 h pulses of chemotherapy with increasing drug concentrations over several months until resistance thresholds of approximately 3-6-fold higher IC50 values were achieved relative to parental cells 37. Cisplatin-resistant MDAH2774, OVSAHO, and IGROV1 ovarian cancer models were generously provided by Dr. Gustavo Baldassarre (National Cancer Institute, Italy) and were previously established through chronic escalating cisplatin exposure 39. PARP inhibitor-resistant ovarian cancer models, including PEO1-derived and COV362 olaparib-resistant cells, were generated through continuous culture in escalating olaparib concentrations ranging from 0.5-10 μM and were generously provided by Dr. Scott Kaufmann (Mayo Clinic, Rochester, MN) 38, 40-42. Mycoplasma testing (SouthernBiotech, Birmingham, AL) was performed regularly to confirm the absence of contamination.

### siRNA knockdown

Transient suppression of CTPS1, HUS1, PRKRA, RAD1, RAD9A, and RHOA was performed using 5 nM control or gene-specific ON-TARGETplus siRNA SMARTpools (Dharmacon, Lafayette, CO) with DharmaFECT transfection reagent #4 (Dharmacon, Lafayette, CO) according to the manufacturer’s protocol. Detailed siRNA information is indicated in Table S1. Knockdown efficiency was validated by RT-qPCR and western blotting 72 h post-transfection.

### RNA isolation and RT-qPCR

Total RNA from breast and ovarian cancer cell lines, benign breast epithelial cells, normal breast tissue, and immortalized fallopian tube epithelial cells was extracted using Trizol reagent (Invitrogen, Thermo Fisher Scientific, Waltham, MA), and cDNA was synthesized with the iScript^TM^ cDNA Synthesis Kit (Bio-Rad Laboratories, Hercules, CA) according to the manufacturer’s protocol. Quantitative PCR was performed with PerfeCTa^TM^ SYBR Green Fast Mix^TM^ (Quanta Biosciences, Gaithersburg, MD). Relative expression of indicated genes was determined using the ΔΔCt method following normalization to 18S and ACTB. Primers were purchased from Integrated DNA Technologies (IDT, Coralville, IA) and their sequences are listed in Table S2.

### Western blotting

Cells were washed with ice-cold PBS and lysed in NETN buffer (150 mM NaCl, 1 mM EDTA, 20 mM pH 8.0 Tris, 0.5% NP-40) supplemented with 1x cOmplete^TM^ Protease Inhibitor Cocktail without EDTA (Roche Applied Science, Indianapolis, IN). Lysates were clarified by centrifugation at 14,000g for 20 min at 4 °C, and protein concentrations were determined using the Pierce BCA Protein Assay Kit (Thermo Fisher Scientific, Waltham, MA). Twenty micrograms of protein were separated on SurePAGE Bis-Tris 4-12% gradient gels (GeneScript, Piscataway, NJ) at 60 mA for 40 min and transferred to PVDF membranes (MilliporeSigma, Burlington, MA) at 550 mA for 30 min. Membranes were blocked for 1 h at room temperature in 5% non-fat milk in 1X TBST and incubated overnight at 4 °C with primary antibodies diluted in blocking buffer. Following washing in 1X TBST, membranes were incubated with HRP-conjugated secondary antibodies for 1 h at room temperature, followed by another set of washing. Blots were imaged on an Odessy Fc Imaging system (LI-COR, Lincoln, NE). Primary antibodies used in this study included CTPS1 (ab244492, rabbit polyclonal, Abcam, Waltham, MA; 1:1000 dilution) and vinculin (ab155120, rabbit polyclonal, Abcam; 1:2000 dilution). HRP-linked anti-rabbit IgG secondary antibody (7074S, Cell Signaling, Danvers, MA) was used at a dilution of 1:2000.

### Proliferation and cell cycle assays

Cell proliferation following siRNA knockdown was determined using crystal violet staining as previous described 37. For cell cycle analysis, cells were fixed in 70% ethanol, stained with propidium iodide/RNase solution (MilliporeSigma, Burlington, MA) and analyzed by flow cytometry (BD FACSCanto, Walpole, MA). Data analysis was performed using FlowJo Version 10.10.0.

### Drug sensitivity assays for 2D cultures

Clinical grade STP938 was provided by Step Pharma (Saint-Genis-Pouilly, France). Indicated cell lines were plated in 96-well plates at a density of 1000 cells per well and treated with a range of STP938 concentrations for 5-7 days. Relative cell numbers were determined by CellTiter-Glo 2D assays (Promega, Madison, WI) according to manufacturer instructions. Data were normalized to DMSO treated controls and IC50 values were calculated using nonlinear regression analysis in GraphPad Prism (v10.6.1).

### Drug sensitivity assays for spheroid cultures

Indicated cell lines were seeded in round-bottom ultra-low adhesion 96-well plates (Corning) at 5,000 cells per well in 100 μL of complete medium. Plates were centrifuged at 120g for 5 min to promote spheroid formation and incubated for 24 h prior to treatment. Spheroids were treated with a range of STP938 concentrations for 7 days. Relative cell viability was determined using CellTiter-Glo 3D assays (Promega), normalized to DMSO-treated controls, and IC50 values were calculated by nonlinear regression analysis in GraphPad Prism (v10.6.1).

### Drug sensitivity assays for 3D bioprinted cultures

Three-dimensional hydrogel cultures were generated using the Rastrum 3D bioprinter (Inventia Life Science, Beaconsfield, Australia). Indicated cell lines were resuspended in the Rastrum cell carrier solution at a density of 1-2x10^6^ cells/mL and bioprinted into 96-well flat bottom plates according to manufacturer guidelines. Cell-laden droplets were immediately encapsulated within Rastrum-compatible bioinks composed of tumor-relevant extracellular matrix components (collagen, laminin, and fibronectin). Bioinks were formulated to have both low (Px02.31P, 1.1 kPa, containing peptides RGD) or high (Px03.28, 3 kPa, containing peptides GFOGER, RGD, DYIGSR) stiffnesses mimicking the mechanical properties of breast and ovarian tumors respectively. Following printing, hydrogel droplets were overlaid with 200 μL of complete medium and cells incubated for 24 h. Cultures were then exposed to a range of STP938 concentrations for 7 days. Cell viability was quantified using CellTiter-Glo 3D assays (Promega) according to the manufacturer’s instructions. Briefly, cultures were equilibrated to room temperature and 100 μL of cell culture medium was removed from each well prior to addition of 100 μL CellTiter-Glo 3D reagent. Plates were shaken at 500 rpm for 10 min to facilitate complete lysis within the hydrogel matrices and incubated at room temperature for an additional 25 min to stabilize the luminescent signal prior to measurement using a luminescence plate reader (Promega). Luminescence values were normalized to DMSO-treated controls, and IC50 values were calculated using nonlinear regression analysis in GraphPad Prism (v10.6.1).

### STP938 activity in PDX models

All animal studies were approved by the Mayo Clinic Institutional Animal Care and Use Committee (IACUC) and conducted under the institutional guidelines. Ovarian cancer PDX models were generated and maintained in female SCID mice as previously described 43-45. For *ex vivo* studies, freshly harvested PDX tumors were dissociated into single-cell suspensions and plated in nano-culture plates (OrganoGenix, Kanagawa, Japan) to allow spheroid formation as previously described 38. Spheroids were treated with vehicle control or STP938 for 3-5 days and cell viability was measured using CellTiter-Glo 3D (Promega). For *in vivo* studies, PDX tumors were established in female SCID mice (n = 3-10) and monitored until measurable tumor burden was achieved. Following tumor establishment, mice were randomized based on ultrasound-indicated tumor size to one of two treatment groups including vehicle control (90% castor oil and 10% benzyl alcohol, Sigma) or STP938 (50 mg/kg, subcutaneous injection, 5 days/week). Tumor size was monitored by weekly ultrasound imaging as previously described 44, 45. Investigators conducting tumor measurements were blinded to treatment allocation. The PDX models minimal information standard (PDX-MI) is detailed in Table S3. Longitudinal tumor area measurements were analyzed on the natural log scale via linear mixed effects models as described previously 46. Fixed effects included time, drug, and the time-by-drug interaction; the spatial (power) covariance structure was used and estimated via restricted maximum likelihood estimation (REML). Goodness of fit was assessed via Akaike and Bayesian Information Criteria (AIC, BIC). Growth trajectories were compared between drug arms via 2-degree of freedom test of coincidence curves.

### CTP quantification by LC-MS

Intracellular CTP levels were quantified using targeted liquid chromatography-mass spectrometry (LC-MS). IGROV1 and IGROV1/CP cells were seeded into 15cm dishes (5-10 x10^6^ cells per dish) and treated the following day with 500nM STP938 or DMSO for 24 or 72 h. At the end of treatment, cells were washed once with cold PBS and collected on ice. Cell pellets were resuspended in 200 μL of 2.5% 5-sulfosalicylic acid (SSA) containing stable isotope-labeled internal standards for CTP (Cambridge Isotope Laboratories, Tewksbury, MA). Samples were mixed thoroughly and subjected to brief probe sonication (3 s, 10% amplitude) to disrupt cells. Lysates were centrifuged at 11,500 g for 2 min at 4 °C, and clarified supernatants were transferred to new tubes for analysis. LC-MS analysis was performed using an Agilent 1260 Infinity II LC system coupled to an Agilent 6150 single quadrupole MSD (Santa Clara, CA). The samples were injected into an Agilent Poroshell 120 EC-C18 column (particle size 2.7 μm, 3.0 x 50 mm) with an InfinityLab Poroshell 120 Bonus-RP, UHPLC guard column (particle size 2.7 μm, 2.1 mm). Temperatures for the auto-sampler were set at 4 °C and the column compartment at 40 °C, respectively. The mobile phase was composed of solvent A (50 mM formic acid in LCMS-grade H2O, adjusted to pH 8.2 with ammonium hydroxide) and solvent B (100% LCMS-grade methanol). The chromatographic gradient was run at a flow rate of 0.3 mL/min as follows: 0-1 min: hold at 20% B; 1-11 min: linear gradient from 20% to 100% B; 11-12 min: hold at 100% B; 12-13 min: linear gradient from 100% to 0% B; 13-20 min: hold at 100% B. The mass spectrometer was operated in selected ion monitoring (SIM), positive ion mode with the capillary voltage set to 3.5 kV, the nozzle voltage set to 2 kV. The sheath gas was held at 250 °C at a flow rate of 10 L/min and the drying gas was held at 300 °C at a flow rate of 5 L/min. The nebulizer pressure was set to 20 psig. The peak area/height was quantified using Agilent ChemStation. The mass to charge ratio (m/z) of the following ions were detected: CTP (m/z 484.2), D5-CTP (m/z 489.2). Peak areas were normalized to the internal standard and expressed relative to DMSO-treated controls. Three independent biological replicates were analyzed for each condition. D5-CTP-only samples were included during LC-MS runs as a quality control to monitor instrument performance, retention time consistency, and signal detection.

### Nucleotide rescue assays

IGROV1 and IGROV1/CP cells were plated in 96-well tissue culture plates and allowed to adhere overnight. The following day, medium was replaced with increasing concentrations of STP938 in the presence or absence of 20 μM exogenous nucleotide supplementation (CTP, ATP, GTP, or UTP; MedChemExpress, Monmouth Junction, NJ). Medium, drug, and nucleotides were refreshed on day 3 and cells were maintained under treatment conditions for a total of 6 days. Cell proliferation was measured using crystal violet assays as previously described 37.

### Statistical analysis

All experiments were performed in at least three independent biological replicates with 2-8 technical replicates per condition depending on the assay. Data are presented as mean ± SEM. One-way or two-way ANOVA with appropriate post hoc corrections were applied unless otherwise indicated. Statistical significance was defined as P < 0.05.

## Results

### Identification of novel molecular dependencies in TNBC cells

**Figure 1:**
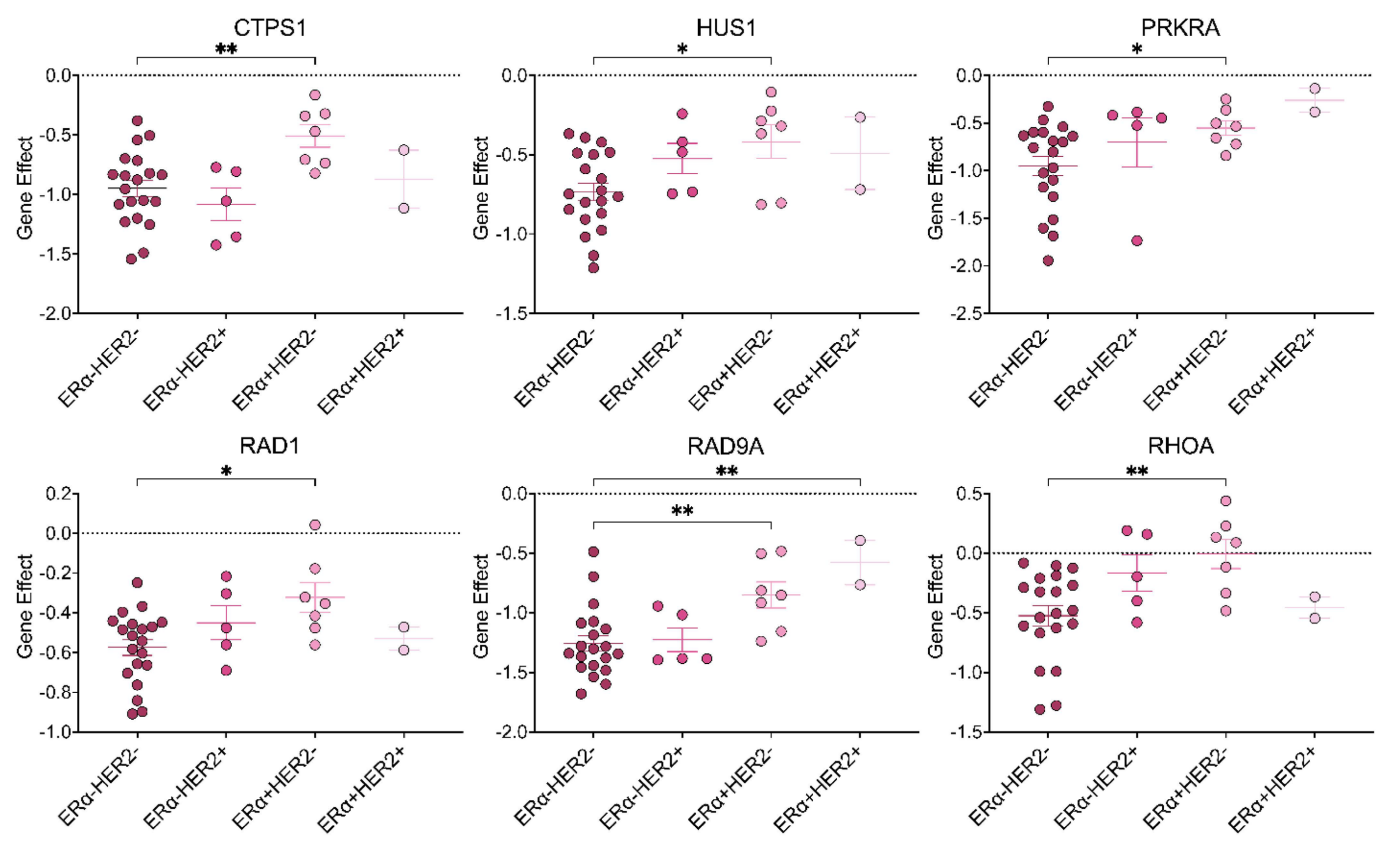
Genetic dependencies in breast cancer cells. Graphical depiction of essentiality scores for indicated genes in over 30 breast cancer cell lines, grouped by specified subtypes, contained within the DepMap Portal. Statistically significant differences between subtypes were identified using one-way ANOVA with correction for multiple comparisons. * P < 0.05, ** P < 0.01.

To identify genes that are more essential for proliferation/viability in TNBC cells relative to non-TNBC cells, we interrogated the results of genome-wide CRISPR knockout screens from the DepMap portal. This analysis revealed six genes (CTPS1, HUS1, PRKRA, RAD1, RAD9A, and RHOA) whose expression was found to be more essential for the proliferation/viability of TNBC cells compared to ERα^+^ cells (Table 1). We subsequently assessed the essentiality scores of these 6 genes in cell lines derived from other breast cancer subtypes (HER2^+^ and ERα^+^/HER2^+^). As shown in Figure 1, there was gene-dependent variability between subtypes, but TNBC cells remained among the most reliant on expression of these 6 genes.

**Figure 2:**
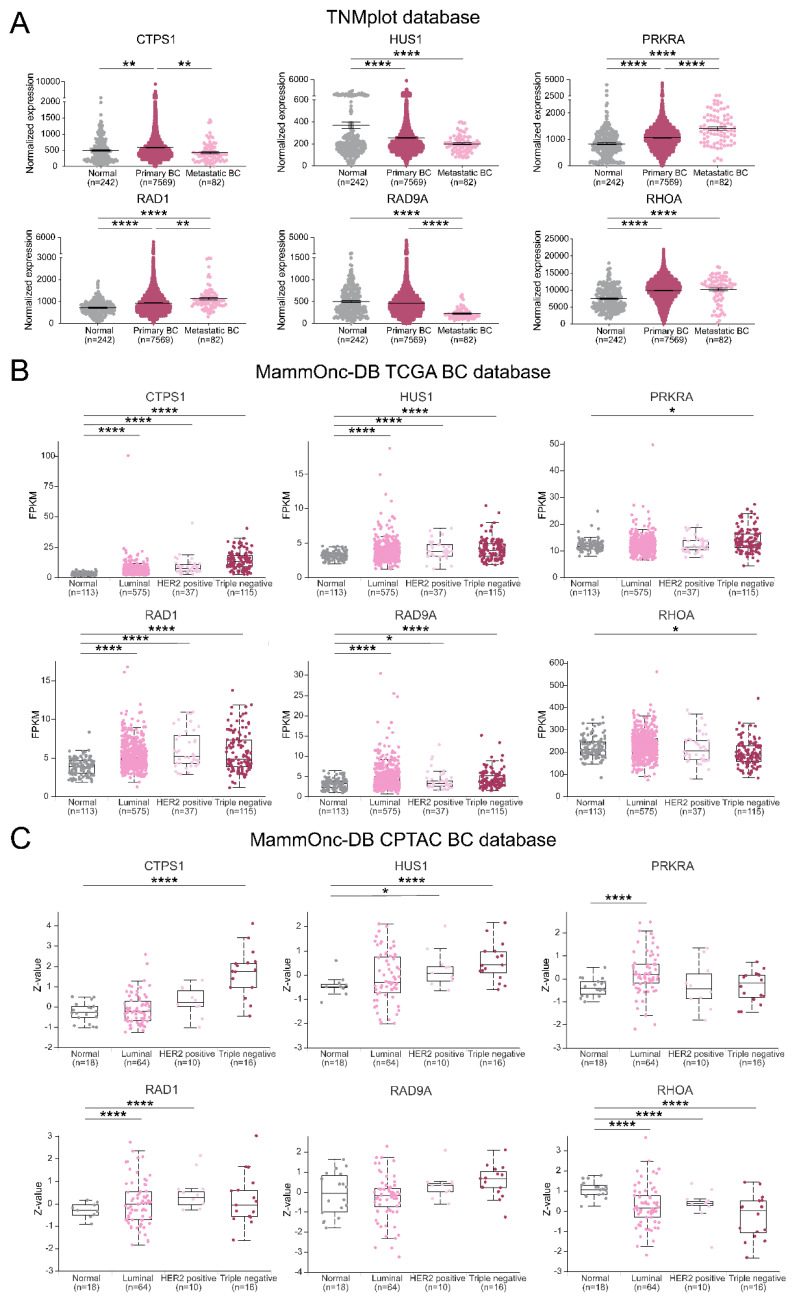
Transcript and protein expression levels of CTPS1, HUS1, PRKRA, RAD1, RAD9A, and RHOA in normal and malignant breast tissue. (A) RNA expression levels of the six genes in normal, primary tumor, and metastatic breast tissues acquired from the TNMplot gene chip database and represent normalized microarray signal intensities (linear scale, arbitrary units). (B) RNA expression across breast cancer subtypes (Luminal, HER2^+^, TNBC) acquired from the MammOnc-DB database within TCGA. (C) Protein abundance for the same genes across breast cancer subtypes obtained from MammOnc-DB within the CPTAC database. Protein abundance is shown as Z-scores representing standardized log2 protein expression across CPTAC samples. Statistically significant differences were identified using one-way ANOVA with correction for multiple comparisons. * P < 0.05, ** P < 0.01, **** P < 0.0001.

We next interrogated the expression profiles of these genes in publicly available datasets at both the RNA and protein levels. Using the TNMplot database, (Figure 2A), CTPS1, PRKRA, RAD1, and RHOA transcripts were significantly higher in primary tumors compared to normal tissue. No differences in RAD9A expression were detected between primary breast tumors and normal breast tissue, while HUS1 was significantly lower in malignant tissue (Figure 2A). The expression levels of PRKRA, RAD1, and RHOA were further elevated in metastatic specimens compared to normal tissue and primary breast tumors (Figure 2A). Conversely, HUS1 and RAD9A exhibited lower mRNA expression in malignant tissue compared with normal tissue (Figure 2A). In the MammOnc-DB TCGA database, 4 of the 6 genes (CTPS1, HUS1, RAD1, and RAD9A) were significantly upregulated at the mRNA level in luminal, HER2^+^ and TN breast tumors compared with normal breast tissue (Figure 2B). PRKRA and RHOA transcripts varied little between cancer subtypes and normal tissue except for slight increases in PRKRA and slight decreases in RHOA in TNBC (Figure 2B). To assess protein levels of these genes in breast tumors, we interrogated the MammOnc-DB CPTAC database. CTPS1 and HUS1 protein abundance were elevated in TNBC compared to normal breast tissue (Figure 2C). No significant differences were detected for PRKRA, RAD1, and RAD9A protein in TNBC whereas RHOA protein expression was lower (Figure 2C). HUS1, PRKRA, RAD1, and RHOA did exhibit significant differences in protein expression levels between other breast cancer subtypes as indicated (Figure 2C).

**Figure 3:**
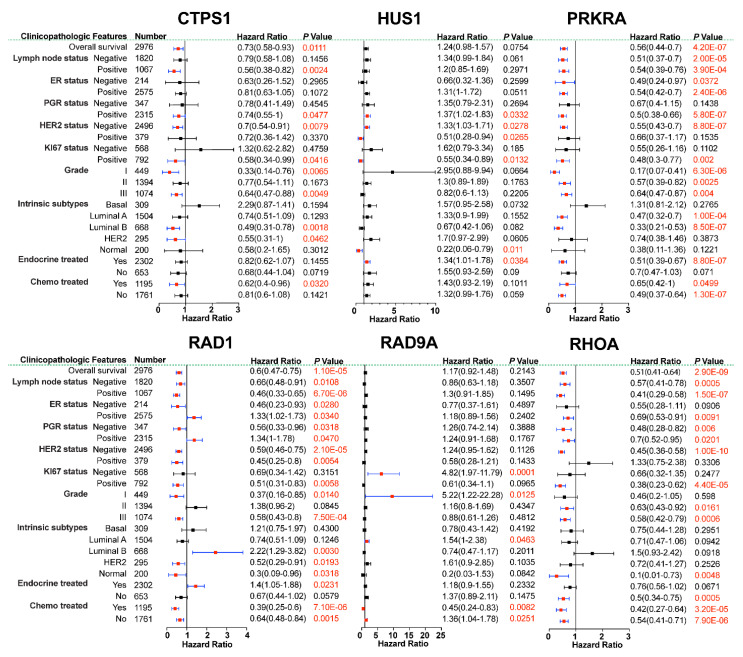
Forest plots summarizing associations between elevated expression of indicated genes with clinicopathologic features and overall survival of breast cancer patients using the Kaplan-Meier Plotter database. Hazard ratios with confidence intervals and P Values are indicated. Hazard ratios were calculated using Cox proportional hazards regression comparing patients with high versus low gene expression (median cutoff). P values were calculated using the log-rank (Mantel-Cox) test. Statistically significant associations are accentuated with colored hazard ratios and confidence intervals as well as red text for P-values.

We next assessed the prognostic relevance of these genes by analyzing their expression in relation to breast cancer clinicopathologic features and overall survival using the Kaplan-Meier Plotter database. Higher expression of each of the 6 genes was generally associated with more aggressive disease characteristics, including advanced tumor grade, lymph node positivity, and a greater likelihood of receiving chemotherapy. More specifically, PRKRA, RAD1, and RHOA exhibited the largest number of statistically significant associations with features such as worse overall survival, lymph node positivity, and elevated Ki67 expression (Figure 3). HUS1 and RAD9A expression exhibited very few significant associations with these features, and for those that were statistically significant, the hazard ratios were numerically small (Figure 3). High CTPS1 expression was correlated with worse overall survival, lymph node positivity, HER2 negativity, Ki67 positivity, and a greater likelihood of receiving chemotherapy (Figure 3). Overall, these findings demonstrate that elevated expression of CTPS1, PRKRA, RAD1, and RHOA are frequently associated with more aggressive breast cancer phenotypes and worse outcomes in this patient cohort.

### Validation of selective essentiality between breast cancer subtypes

**Figure 4:**
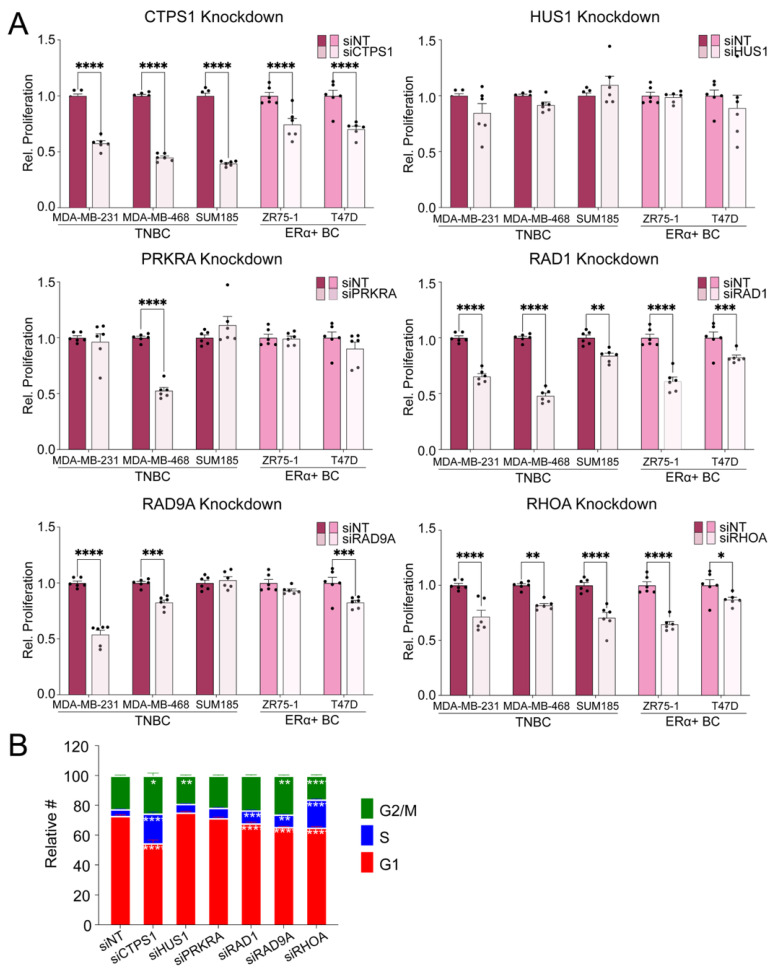
Impact of gene-specific knockdown on cell proliferation and cell cycle progression. (A) Relative proliferation rates of indicated cell lines following siRNA mediated gene suppression relative to a non-targeting siRNA control across a panel of triple negative and ERα^+^/HER2^-^ breast cancer cell lines. (B) MDA-MB-231 cell cycle profiles following knockdown of indicated genes as quantified via propidium iodide staining and flow cytometry. Data are represented as mean ± SEM. Two-way ANOVA with correction for multiple comparisons was used to identify significant differences. * P < 0.05, ** P < 0.01, *** P < 0.001, **** P < 0.0001.

Having integrated selective dependency patterns from DepMap with differential gene expression, clinicopathologic features, and survival outcomes in breast cancer patient cohorts, we sought to validate the reliance of TNBC cells on expression of these 6 genes for growth/survival. CTPS1, HUS1, PRKRA, RAD1, RAD9A, and RHOA gene-specific siRNA SMARTpools were transfected into three TNBC cell lines (MDA-MB-231, MDA-MB-468, and SUM185) and two ERα^+^/HER2^-^ cell lines (ZR75-1 and T47D). Transfection conditions were optimized and successful knockdown was confirmed by RT-qPCR (Figure S1A) and western blotting (Figure S1B). The effects of knockdown on cell proliferation were assessed using crystal violet staining. Knockdown of CTPS1, RAD1, and RHOA, significantly inhibited proliferation in all 5 cell lines compared to the non-targeting control siRNA (Figure 4A). RAD9A was found to suppress two of the three TNBC models and one ERα^+^/HER2^-^ model (Figure 4A). Suppression of PRKRA expression only inhibited MDA-MB-468 cells while knockdown of HUS1 had no significant effect on any of the cell lines tested (Figure 4A). When considering the magnitude of these effects between the two subtypes of breast cancer cell line models, only knockdown of CTPS1 was shown to more inhibitory in all three TNBC models compared to ERα^+^/HER2^-^ models (Figure 4A).

To determine the impact of knockdown on cell cycle progression, propidium iodide staining followed by flow cytometry was performed using MDA-MB-231 cells harvested 72 h after siRNA transfection. Consistent with the proliferation data, knockdown of HUS1 and PRKRA in MDA-MB-231 cells had little to no effect on the cell cycle (Figure 4B). Following suppression of RAD1 and RAD9A, significantly more cells were observed in S phase with concomitant decreases in the proportion of cells in G1 (Figure 4B). Significant accumulation of cells in G2/M was also observed in RAD9A depleted cells (Figure 4B). Knockdown of CTPS1 and RHOA induced significant S phase arrest, however, the downstream effects on other cell-cycle phases differed between the two targets (Figure 4B). RHOA inhibition led to a reduction in both G1 and G2/M phases while CTPS1 knockdown resulted in a significant decrease in G1 alongside an increase in G2/M (Figure 4B).

Among the six candidate genes identified through integrated dependency, transcriptomic, and proteomic analyses, CTPS1 was prioritized for further investigation based on its consistent dependency across TNBC models, elevated RNA and protein expression in malignant breast tumors including TNBC relative to normal tissue, association with aggressive clinicopathologic features and poor patient outcomes, established role in nucleotide biosynthesis, and limited investigation of its therapeutic utility. Collectively, these observations suggested that CTPS1 represented a biologically and clinically relevant candidate for further study.

### CTPS1 expression in ovarian cancer

**Figure 5:**
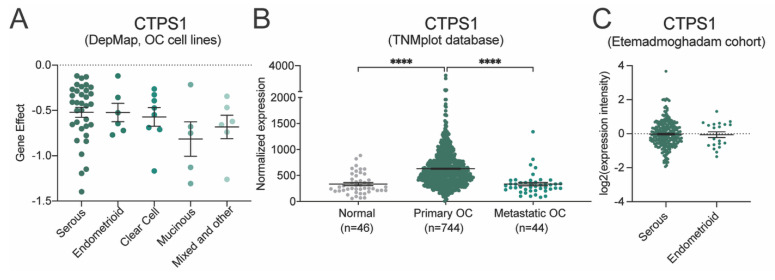
CTPS1 essentiality and expression patterns in ovarian cancer. (A) Essentiality scores for CTPS1 across ovarian cancer cell lines included in the DepMap Portal stratified based on histologic subtype. (B) CTPS1 mRNA expression in normal ovarian tissue, primary ovarian cancers, and metastatic ovarian tissues contained within the TNMplot gene chip database and represent normalized microarray signal intensities (linear scale, arbitrary units). (C) CTPS1 mRNA expression in serous and endometrioid ovarian tumors based on the Etemadmoghadam *et al*. cohort. Statistically significant differences between subtypes were assessed using one-way ANOVA with correction for multiple comparisons. **** P < 0.0001.

In parallel to the breast cancer studies, we also discovered that CTPS1 was a growth promoting factor in ovarian cancer cell lines (gene effect scores < 0), some of which were reliant on its expression for survival (gene effect scores < -1), according to DepMap (Figure 5A). No significant differences in CTPS1 essentiality were detected between the various histological subtypes (Figure 5A). Like that of breast cancer, CTPS1 mRNA expression was elevated in malignant vs. normal ovarian tissue (TNMplot database) for which histologic subtypes are not indicated (Figure 5B). Additionally, no significant differences were observed in CTPS1 expression between metastatic ovarian cancer and normal tissue (Figure 5B). Using a separate patient cohort (Etemadmoghadam *et al*. dataset) 35, no differences in CTPS1 expression were found between serous (low- and high-grade) and endometrioid ovarian (Figure 5C).

### CTPS1 mRNA expression in breast and ovarian cancer cell lines and drug-resistant models

**Figure 6:**
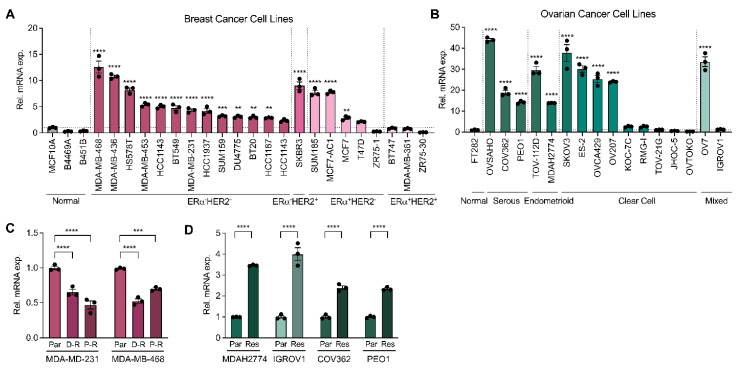
CTPS1 expression in breast and ovarian cancer cell lines. RT-PCR analyses indicating the relative expression levels of CTPS1 in a panel of (A) breast and (B) ovarian cancer cell lines compared to benign cells and normal tissue. Data are normalized to MCF10A cells in panel A and FT282 cells in panel B. CTPS1 expression levels in chemotherapy-resistant, and PARP inhibitor-resistant isogenic (C) breast and (D) ovarian cancer cell line models following normalization to their respective parental controls. Par, parental cells; D-R, doxorubicin-resistant cells; P-R, paclitaxel-resistant cells. Data are represented as mean ± SEM from n = 3 replicates. Significance was assessed using two-way ANOVA with correction for multiple comparisons. * P < 0.05; **** P < 0.0001.

Using a panel of available breast and ovarian cancer cell lines, CTPS1 mRNA expression was found to be variable, but overall increased, relative to non-malignant control cell lines and tissues including benign breast epithelial cells (MCF10A), normal breast tissue (B4469A and B451B) and immortalized normal fallopian tube epithelial cells (FT282) (Figure 6A-B). Overall, TNBC cell lines exhibited the highest levels of CTPS1 expression, while ERα^+^/HER2^+^ models had the lowest levels with minimal to no significant differences from benign cells or normal tissue (Figure 6A). Ovarian cancer cell lines were found to be similarly heterogeneous for CTPS1 expression, with serous and endometrioid models showing robustly increased levels compared to FT282 cells (Figure 6B). Clear cell and mixed histology cell lines showed greater variability, with some exhibiting very high CTPS1 expression and others showing little to no difference from FT282 control cells (Figure 6B). Doxorubicin-resistant (D-R) and paclitaxel-resistant (P-R) TNBC models were found to exhibit decreased CTPS1 expression relative to their isogenic parental cells (Figure 6C). In contrast, cisplatin-resistant (MDAH2774, IGROV1, PEO1) and/or PARP inhibitor-resistant (COV362, PEO1) ovarian cancer cell lines were found to express higher levels of CTPS1 compared to their respective isogenic parental controls (Figure 6D).

### CTPS1 inhibition as a targeted therapeutic strategy

In partnership with Step Pharma, which developed the first-in-class CTPS1 small molecule inhibitor, STP938 (dencatistat), we evaluated the effect of STP938 on breast and ovarian cancer cell lines. Clinically achievable nanomolar IC50s were found for many breast cancer cells and all ovarian cancer models tested, including therapy-resistant models (Table 2). Interestingly, little to no inhibitory activity was observed in multiple non-TNBC breast cancer cell lines (Table 2). Consistent with on-target CTPS1 inhibition, short-term STP938 treatment induced S-phase cell cycle arrest in both parental and therapy-resistant TNBC and ovarian cancer models (Figure S2) as was the case following transfection with CTPS1 specific siRNAs.

**Figure 7:**
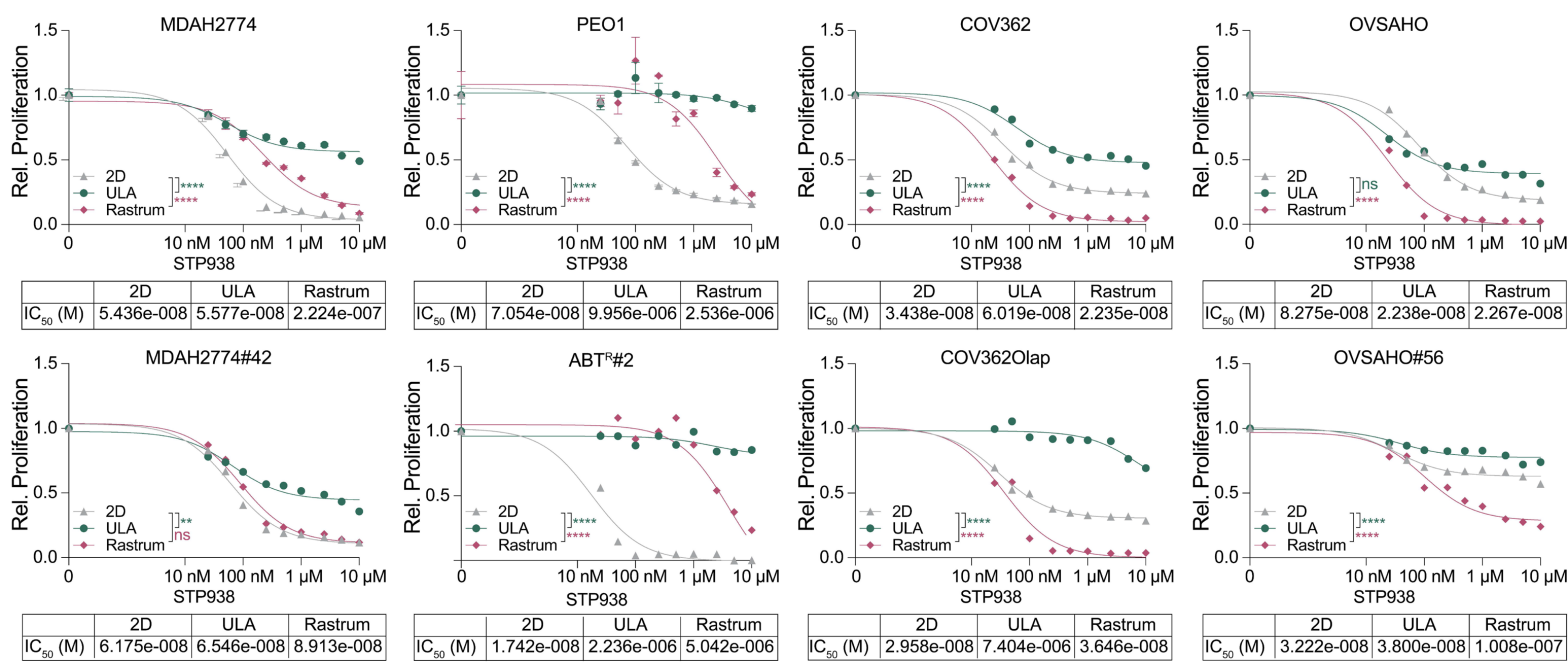
STP938 sensitivity across 2D, ULA spheroid, and 3D bioprinted ovarian cancer models. Dose-response curves for cisplatin- and PARP inhibitor-sensitive ovarian cancer cell lines (top row) and their respective isogenic cisplatin-resistant (MDAH2774, OVSAHO, ABT^R^#2) or PARP inhibitor-resistant (COV362Olap, ABT^R^#2) derivatives (bottom row), treated with increasing concentrations of STP938 in 2D monolayer culture (grey triangles), ultra-low attachment (ULA) spheroid culture (green circles), and Rastrum 3D bioprinted hydrogel cultures (pink diamonds). Relative proliferation was measured after 7 days for 2D and ULA cultures and after 14 days for 3D bioprinted models, normalized to DMSO controls, and plotted as mean ± SEM. Data represent n = 3-6 replicates per condition. Significant differences from 2D cultures were assessed using two-way ANOVA with correction for multiple comparisons. ** P < 0.01, **** P < 0.0001.

**Figure 8:**
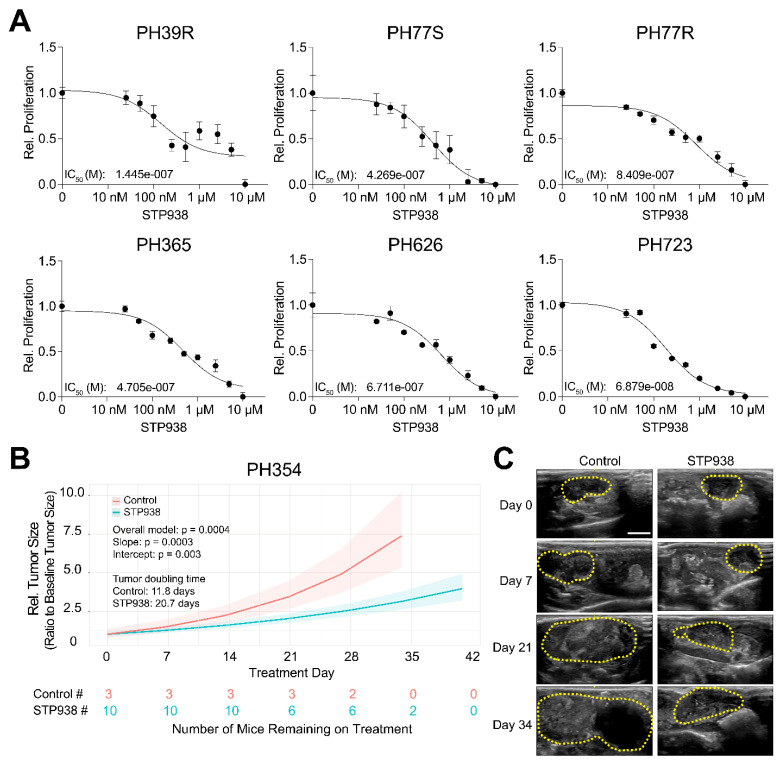
Efficacy of STP938 in ovarian cancer PDX models. (A) Dose-response curves for STP938 in six ovarian cancer PDX models, including both therapy-sensitive and PARP inhibitor-resistant tumors, assessed in *ex vivo* spheroid cultures. Relative cell viability was normalized to vehicle-treated controls and IC50 values are indicated for each model. Data represent mean ± SEM from n = 4 replicates. (B) Tumor growth curves generated from ultrasound-based measurements (cm^2^) of the PH354 ovarian cancer PDX model treated with vehicle control (n = 3) or STP938 (50 mg/kg, n = 10). Tumor size at each timepoint was normalized to starting tumor size prior to treatment initiation and plotted as relative values throughout the course of the experiment. Shaded areas represent SEM. Significant differences between treatment groups were determined using a linear mixed-effects model for repeated measures and p-values are indicated for the overall model, slope of the lines, and intercept of the lines. Tumor doubling time was also calculated. (C) Representative ultrasound images of a vehicle treated and STP938 treated mouse showing intraperitoneal tumors at indicated time points. Yellow dashed lines define the tumor area. Scale bar = 5 mm.

Given that ovarian tumors frequently induce the formation of ascites fluid in which tumor cells grow in the absence of attachment to a basement membrane, we next examined the activity of STP938 in spheroid cultures generated using ultra-low-attachment (ULA) plates. Across matched cisplatin-sensitive (MDAH2774, OVSAHO, PEO1) and -resistant (MDAH2774#42, OVSAHO#56, ABT^R^#2) cell lines, as well as PARPi-sensitive (COV362, PEO1) and -resistant (COV362Olap, ABT^R^#2) models, ULA spheroids exhibited significantly reduced sensitivity to STP938 compared with 2D monolayer cultures (Figure 8). Interestingly, some cell lines were unaffected by STP938 even at concentrations above 10 µM when grown in ULA conditions (Figure 7).

We further examined STP938 responses in 3D bioprinted cultures generated using Rastrum hydrogels, which better recapitulate the architectural and extracellular-matrix features of native tumors 47. For a subset of models (OVSAHO, OVSAHO#56, COV362, and COV362Olap), 3D printed cultures were significantly more sensitive to STP938 than 2D cultures as reflected by left-shifted dose response curves (Figure 7). Cisplatin-sensitive and -resistant MDAH2774 models exhibited similar levels of sensitivity in 3D hydrogels as in 2D cultures (Figure 7). The sensitivity of PEO1 and ABT^R^#2 cells to STP938 in 3D cultures were intermediate to that of 2D monolayer cultures and ULA spheroid cultures (Figure 7). These findings demonstrate that for most ovarian cancer cell lines, STP938 elicits anti-neoplastic activity across multiple culture systems.

### STP938 activity in ovarian cancer PDX models

To determine whether the anti-neoplastic activity of STP938 observed in established ovarian cancer cell line models also occurs in patient-derived tumor models, we evaluated the effects of this drug using both *ex vivo* and *in vivo* approaches. For *ex vivo* studies, six ovarian cancer PDX models (PH39R, PH77S, PH77R, PH365, PH626, and PH723) were expanded in mice, resected, mechanically and enzymatically digested into single cell suspensions, and cultured as spheroids. Spheroids included both human tumor cells and any associated intra-tumoral mouse stromal cells. Among these models, PH77S was derived from a treatment-naïve tumor previously shown to respond to platinum-based therapy, whereas PH39R and PH77R represent related models that were developed to be PARP inhibitor-resistant following repeated niraparib exposure *in vivo* 38, 45, 48, 49. The remaining models (PH365, PH626, and PH723) were established from high-grade serous or clear cell ovarian tumors obtained from treatment-naïve patients with advanced stage disease, thereby representing additional clinically relevant ovarian cancer subtypes within the PDX cohort. In all six models, STP938 treatment resulted in dose-dependent inhibition of spheroid viability with IC50 values in the nanomolar range (Figure 8A). We next sought to evaluate the *in vivo* efficacy of STP938. We chose to use a seventh PDX model derived from a patient with high-grade serous primary peritoneal carcinoma following neoadjuvant therapy (PH354) given that this closely mimics the clinical situation of the ongoing clinical trial. Additionally, this model exhibits robust tumor engraftment and growth characteristics and has proven to enable reliable assessment of therapeutic response to a variety of drugs *in vivo*. STP938 treatment was well tolerated with no overt signs of toxicity or weight loss observed during the study. Pharmacologic inhibition of CTPS1 significantly suppressed tumor growth relative to vehicle control treated mice (Figure 8B-C). These findings further demonstrate the efficacy of STP938 in a highly aggressive and therapy resistant patient-derived model, further supporting the pre-clinical and clinical development of this drug.

### Intracellular CTP levels following CTPS1 inhibition

**Figure 9:**
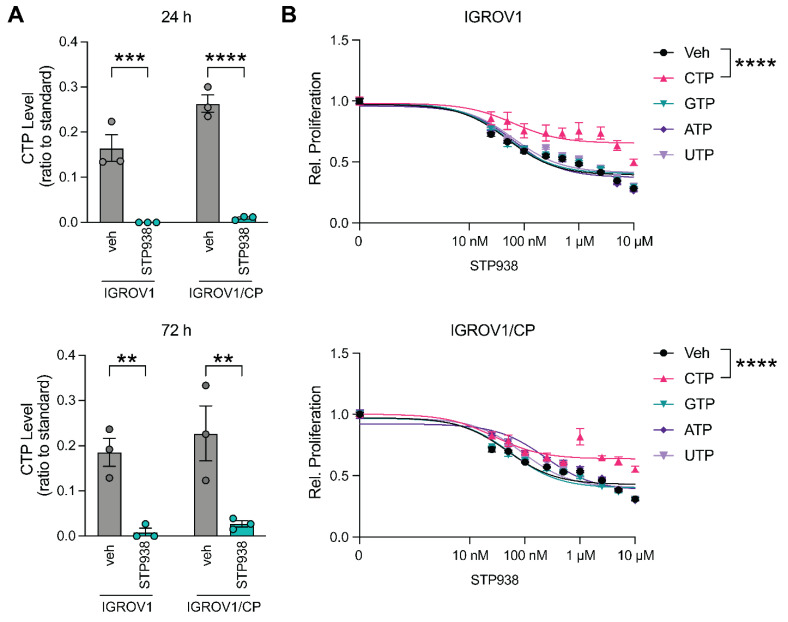
On-target activity of STP938 in cisplatin-sensitive and cisplatin-resistant ovarian cancer cells. (A) Intracellular CTP abundance was quantified by LC-MS in IGROV1 (cisplatin-sensitive) and IGROV1/CP (cisplatin-resistant) cells following treatment with 500 nM STP938 for 24 or 72 h. Data are presented as mean ± SEM from n = 3 biological replicates. Significant differences from vehicle treatment were assessed using two-way ANOVA with correction for multiple comparisons. ** P < 0.01, *** P < 0.001, **** P < 0.0001. (B) IGROV1 and IGROV1/CP cells were treated with increasing concentrations of STP938 in the presence or absence of 20 μM exogenous nucleotides (CTP, ATP, GTP, or UTP) for 6 days with treatment refreshed on day 3. Data are presented as mean ± SEM from n = 4 biological replicates. Significant differences between vehicle and nucleotide rescue conditions were assessed using one-way ANOVA with correction for multiple comparisons. **** P < 0.0001.

To determine whether STP938 achieves on-target biochemical inhibition of CTPS1 in cisplatin-sensitive and -resistant ovarian cancer models at clinically relevant concentrations, we quantified intracellular CTP levels by LC-MS in IGROV1 and IGROV1/CP cells following drug treatment. Because CTPS1 catalyzes the rate-limiting step in the conversion of UTP to CTP, effective target inhibition should result in measurable depletion of intracellular CTP pools. As shown in Figure 9, treatment with 500 nM STP938 ablated intracellular CTP in both IGROV1 and IGROV1/CP cells 24 h after drug treatment. At the 72 h timepoint, CTP levels remained very low but showed evidence of slight recovery (Figure 9A). These findings demonstrate that STP938 achieves potent suppression of CTP synthesis after a single treatment administration in ovarian cancer cells and provide biochemical evidence of robust target engagement.

To further determine whether reduced CTP availability contributes directly to the anti-neoplastic effects of STP938, IGROV1 and IGROV1/CP cells were treated with increasing concentrations of STP938 in the presence or absence of exogenous nucleotides. Supplementation with exogenous CTP significantly rescued STP938-mediated growth inhibition in both cell lines, while ATP, GTP, and UTP supplementation had no effect (Figure 9B). These findings support the conclusion that selective depletion of intracellular CTP pools contributes directly to impaired proliferation following CTPS1 inhibition and further support the on-target activity of STP938.

### Association of CTPS1 and CTPS2 expression with STP938 sensitivity

**Figure 10:**
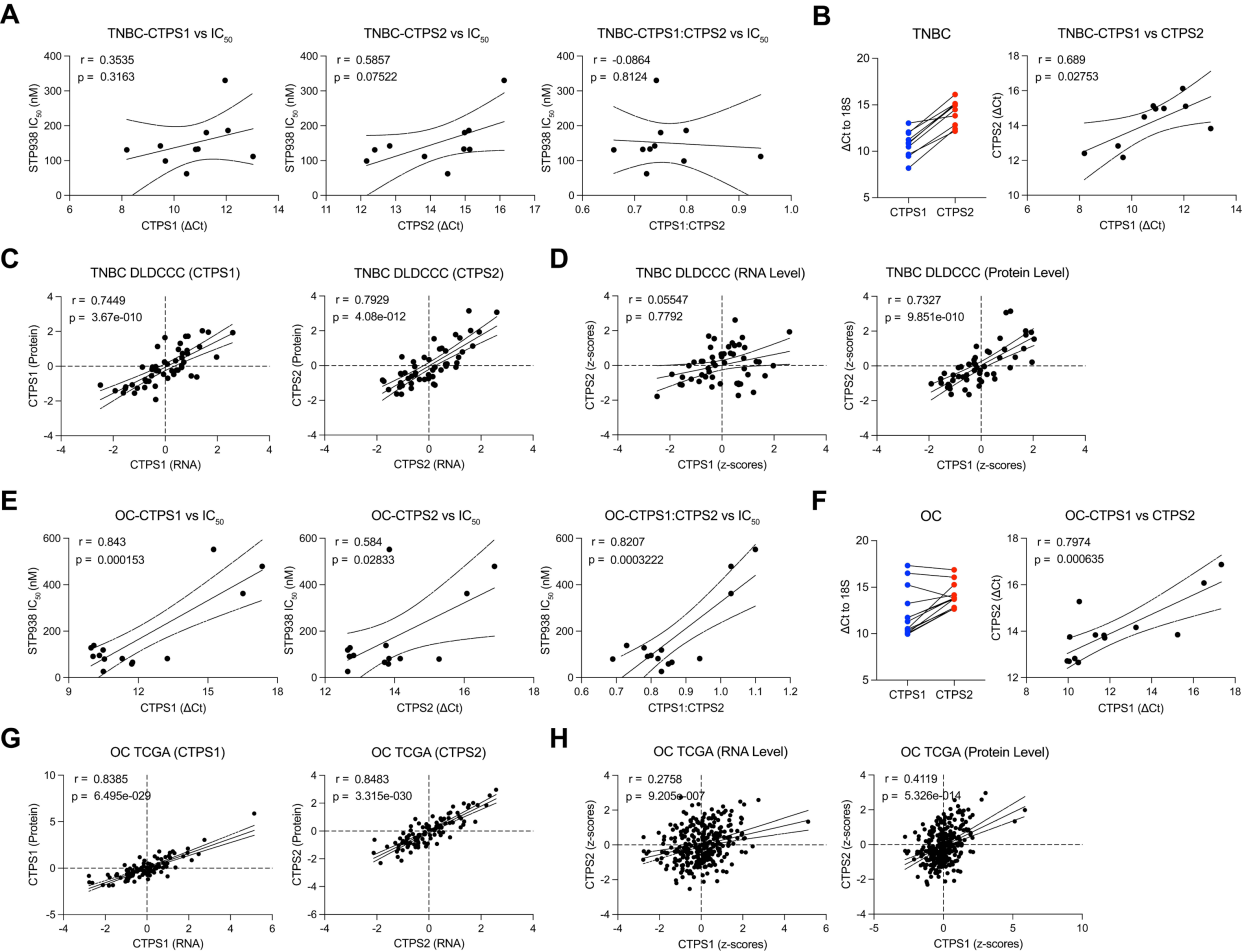
Associations of CTPS1 and CTPS2 expression with STP938 sensitivity in TNBC and ovarian cancer cell lines and tumors. (A) Scatter plots depicting correlation between CTPS1 ΔCt, CTPS2 ΔCt, or the CTPS1:CTPS2 ΔCt ratio, and STP938 IC50 values in TNBC cell lines. (B) Paired CTPS1 and CTPS2 ΔCt values for TNBC cell lines are shown in the left panel, with lines connecting values derived from the same cell line. Association of CTPS1 and CTPS2 mRNA expression among TNBC models are shown to the right. (C) Correlation analyses of CTPS1 and CTPS2 mRNA expression with their respective protein abundance in TNBC tumors contained within the DLDCC dataset. Scatter plots show z-score-normalized RNA vs CTPS protein abundance. (D)Correlation analyses of CTPS1 and CTPS2 mRNA (left) and protein (right) in TNBC tumors within the DLDCC dataset. (E) Scatter plots showing the relationship between CTPS1 ΔCt, CTPS2 ΔCt, or the CTPS1:CTPS2 ΔCt ratio and STP938 IC50 values in ovarian cancer cell lines. (F) Paired CTPS1 and CTPS2 ΔCt values for ovarian cancer cell lines are shown in the left panel, with lines connecting values derived from the same cell line. Association of CTPS1 and CTPS2 mRNA expression among ovarian cancer models are shown to the right. (G) Correlation analyses of CTPS1 and CTPS2 mRNA expression with their respective protein abundance in ovarian tumors contained within the TCGA dataset. (H) Correlation analyses of CTPS1 and CTPS2 mRNA (left) and protein (right) in ovarian tumors within the TCGA dataset. For all correlation plots, linear regression analyses were performed, and correlation coefficients (r) and p-values are displayed within each panel. ΔCt values represent transcript levels of indicated gene following normalization to 18S rRNA. Higher ΔCt values indicate lower CTPS1 or CTPS2 mRNA expression.

In addition to CTPS1, a second gene, CTPS2, is also able to catalyze the synthesis of CTP. We therefore determined if a correlation exists between expression of the CTPS isoforms and STP938 efficacy in TNBC and ovarian cancer cell lines. As shown in Figure 10A, neither CTPS1 nor CTPS2 transcript levels were associated with drug sensitivity in TNBC models, although higher expression of both genes trended toward increased sensitivity to STP938. The ratio of CTPS1:CTPS2 also showed no association with STP938 responsiveness (Figure 10A). CTPS1 and CTPS2 expression levels were found to correlate with one another in the tested panel of TNBC cells (Figure 10B). To determine whether mRNA expression reflected protein abundance in TNBC, we analyzed the DLDCC TNBC dataset 34. CTPS1 and CTPS2 RNA levels each showed strong and highly statistically significant correlations with their respective protein abundances (Figure 10C). CTPS1 and CTPS2 RNA levels were weakly associated with one another in this dataset (Figure 10D, left) and their protein abundances were found to be significantly correlated (Figure 10D, right).

In contrast, ovarian cancer cell lines showed robust and statistically significant correlations between CTPS1 and CTPS2 expression, as well as the ratio of CTPS1:CTPS2, with STP938 IC50 values. Specifically, higher expression of both genes, as reflected by lower ΔCt values, was strongly associated with lower IC50 values (Figure 10E). Notably, ovarian cancer cell lines exhibited greater variability in STP938 IC50 values compared with TNBC models (Figure 10A&E). As in TNBC, CTPS1 and CTPS2 mRNA expression levels were tightly correlated with each other in the tested ovarian cancer cell line panel (Figure 10F). Analysis of the TCGA ovarian cancer dataset further demonstrated strong correlations between CTPS1 RNA and protein levels, between CTPS2 RNA and protein levels, and between CTPS1 and CTPS2 expression at both the RNA and protein levels (Figure 10G-H). Taken together, these finding demonstrate that mRNA expression for CTPS1 and CTPS2 may be a valid surrogate for protein levels in TNBC and ovarian cancer, and that isoform specific expressions are predictive of response to STP938 for ovarian cancer.

## Discussion

As outlined here, we identified CTPS1 as a critical molecular dependency in TNBC and ovarian cancer cell lines through interrogation of the DepMap Portal. We validated the reliance of many TNBC and ovarian cancer cells on CTPS1 via siRNA-mediated knockdown and assessment of cell proliferation and cell cycle profiling. We demonstrate using multiple publicly available patient databases that CTPS1 expression is elevated in malignant tissue relative to non-malignant and benign models. We also show that elevated CTPS1 mRNA levels are associated with more aggressive tumor characteristics and worse patient outcomes. Notably, transcript and protein abundance were not fully concordant for all candidate genes identified in our analyses. For example, RHOA mRNA expression was elevated in some patient datasets whereas corresponding protein abundance was not similarly increased in TNBC tumors. Such discrepancies are common and may reflect post-transcriptional regulation, differences in translational efficiency, altered protein stability, or enhanced protein turnover 50. These observations further highlight the importance of integrating both transcriptomic and proteomic datasets when prioritizing candidate molecular dependencies for functional validation. We discovered that CTPS1 expression is further elevated in ovarian cancer models of chemotherapy- and PARPi-resistance. Using the first-in-class CTPS1 selective inhibitor, STP938, we demonstrate robust anti-cancer activity of this small molecule across a panel of TNBC and ovarian cancer cells and confirm its on-target activity by demonstrating abolition of intracellular CTP within 24 h of drug treatment. Overall, our findings establish CTPS1 as a potential therapeutic vulnerability in TNBC and ovarian cancer, supporting the continued pre-clinical and clinical of STP938. These findings align with growing evidence that dysregulated nucleotide metabolism represents a hallmark of highly proliferative, therapy-resistant cancers 27, 51, 52. Prior studies have implicated CTPS1 in cell-cycle progression, replication stress tolerance, immune cell proliferation, and dependency in some solid tumors including TNBC 24, 53-57. However, the therapeutic activity of the selective CTPS1 inhibitor STP938 has not previously been evaluated in TNBC or ovarian cancer models. The results presented here expand our understanding of CTPS1 in TNBC and ovarian cancer and provide the first demonstration of STP938 efficacy in models of these two forms of cancer.

The identification of CTPS1 as a dependency in TNBC is particularly important given the lack of targeted therapies and alternative treatment approaches for this subtype of cancer. Unlike ERα^+^ or HER2^+^ breast tumors, which derive great benefit from endocrine therapies or HER2 targeting antibodies and small molecules respectively, the treatment of TNBC remains heavily reliant on chemotherapy. TNBC patients have extremely poor outcomes when they experience a recurrence within 5-years of diagnosis, or present with *de novo* resistant forms of the disease 4, 12-15, 37. Similarly, ovarian cancer remains one of the most lethal forms of cancer due to frequent and rapid recurrence following curative intent treatment 18 with limited alternative maintenance therapies 17, 19, 20. Thus, the discovery that many TNBC and ovarian cancer cell lines and tumors exhibit elevated CTPS1 expression, and have become reliant on its activity for growth, suggests that STP938 may be a tractable therapeutic for these diseases.

Critically, we found that CTPS1 expression is elevated in some chemotherapy- and PARPi-resistant ovarian cancer models which concomitantly display increased susceptibility to CTPS1 inhibition. These results suggest that CTPS1 expression may be part of a stress adaptive program that supports cell survival and eventual tumor outgrowth following standard-of-care therapy. Importantly, our findings do not establish CTPS1 as a direct driver of chemotherapy or PARP inhibitor resistance but rather support a model in which increased CTPS1 dependency reflects elevated nucleotide demand associated with proliferation and DNA repair in therapy-stressed tumor cells. Interestingly, increased CTPS1 expression was more consistently observed in therapy-resistant ovarian cancer models than in resistant TNBC models. This difference may reflect lineage-specific metabolic dependencies that emerge during therapeutic adaptation. Therapy-resistant ovarian tumors frequently exhibit enhanced replication stress and increased reliance on nucleotide biosynthesis pathways to sustain DNA repair and survival 58, 59. In contrast, resistant TNBC models may utilize more heterogeneous adaptive mechanisms, including metabolic plasticity, altered apoptotic signaling, epithelial-to-mesenchymal transition programs, or increased reliance on nucleotide salvage pathways 60-62. Differential CTPS2 expression or compensatory pathway redundancy may also contribute to these lineage-specific differences and may partially explain the stronger relationship between CTPS1/CTPS2 expression and STP938 sensitivity observed in ovarian cancer models. However, the mechanisms driving CTPS1 overexpression in advanced and therapy-resistant cancers remain unclear. According to TCGA data, amplification of CTPS1 is infrequent in primary TNBC and ovarian tumors and additional cohorts of recurrent/metastatic tumors are needed to determine if amplification during later stage disease contributes to elevated expression. Previous studies have suggested that CTPS1 is transcriptionally regulated by oncogenic signaling pathways such as MYC, P53, YBX1, metabolic rewiring, or stress-induced activation of nucleotide synthesis programs 23, 56, 63-66. Additionally, elevated CTPS1 expression may involve epigenetic reprograming or stress-adaptive transcriptional programs. Future studies aimed at interrogating the contribution of MYC-, YBX1-, or stress-associated signaling pathways to CTPS1 upregulation in therapy-resistant cancer models will be important to better define the mechanisms underlying CTPS1 dependency.

Using the STP938 small molecule, we pharmacologically validated that many TNBC and ovarian cancer cells are highly reliant on CTPS1. Although CTPS1 and CTPS2 catalyze the same biochemical reaction, our data support prior evidence that CTPS2 is unable to fully compensate for loss of CTPS1 expression and activity, given the near complete loss of CTP following STP938 treatment 24, 54. This functional non-redundancy likely explains why STP938 is able to elicit such robust anti-neoplastic activity despite the fact that it does not engage CTPS2 at the IC50s identified here 23. Indeed, the present studies demonstrate that STP938 exhibits nanomolar potency across TNBC and ovarian cancer models, whereas ER^+^ breast cancer cells are significantly more resistant, underscoring the specificity of CTPS1 dependency. Interestingly, several ovarian cancer cell lines cultured within 3D bioprinted hydrogels exhibited greater sensitivity to STP938 than corresponding 2D monolayer cultures. These observations suggest that extracellular matrix composition and biomechanical properties may influence CTPS1 dependency and therapeutic response. Matrix stiffness and cell-ECM interactions are known to alter proliferation dynamics, replication stress responses, metabolic activity, and therapeutic sensitivity in solid tumor models 67-69. It is therefore possible that the physiologically relevant microenvironment created by the 3D hydrogel system enhances reliance on nucleotide biosynthesis pathways in a subset of ovarian cancer models, thereby increasing sensitivity to CTPS1 inhibition. Importantly, we observed similar anti-neoplastic activity in seven independent ovarian cancer PDX models, where STP938 significantly inhibited PDX cell viability *ex vivo* and suppressed tumor growth *in vivo*, including in models with acquired PARP inhibitor resistance. Previous studies have also implicated the reliance of some solid tumor cancer cell lines on CTPS1 expression 28, 54, 57, including TNBC 56. However, prior to this report, there was no published literature related to the efficacy of STP938 in any solid tumor model. Our findings further support the recently activated clinical trial studying this drug in advanced solid tumors, including breast and ovarian cancer (NCT06297525).

As shown by cell cycle profiling, STP938 induces S-phase cell cycle arrest which is consistent with the observation that intracellular CTP levels plummet in cancer cells following exposure to this drug. Cells actively undergoing DNA synthesis encounter difficulties with DNA duplication in the absence of CTP. Given these findings, it is curious why some cancer cells are largely able to persist and divide in the presence of STP938. The partial recovery of intracellular CTP pools observed at later timepoints could simply be reflective of drug metabolism or degradation over time since these studies were performed following a single administration of the compound. However, it is also possible that these observations are reflective of adaptive metabolic compensation that occurs in response to CTPS1 inhibition such as activation of the pyrimidine salvage pathway. These findings also highlight the necessity of sustained target suppression that will need to be critically evaluated and optimized in clinical trials to ensure durable depletion of intracellular CTP pools and maximize anti-neoplastic efficacy.

Along these lines, our findings raise the possibility of several rational combinatorial treatment approaches. Given that STP938 effectively depletes intracellular CTP levels, an imbalance in nucleotide pools would result. Such imbalances are known to cause replication-fork stalling, DNA breaks, and activation of DNA damage and repair mechanisms, frequently leading to apoptosis 70-72. Therefore, pairing STP938 with DNA-damaging agents, replication stress inducers, or DNA repair inhibitors may yield synergistic effects 55, 64, 65. This strategy could be especially valuable in resistant tumors, where CTPS1 is most highly expressed. Indeed, we demonstrate here that STP938 monotherapy is highly effective in chemotherapy- and PARPi-resistant models of TNBC and ovarian cancer. Further, STP938 may prove to be an ideal drug partner for PARPi, even in patients whose tumors are homologous recombination proficient where PARPi are known to be largely ineffective 73, 74.

Given the importance of CTPS1 for the proliferation of activated T- and B- cells as part of normal immune system function 24, 53, it will be critical to determine how STP938 impacts anti-tumor immunity, and whether checkpoint inhibitors or other immune-targeting strategies could enhance efficacy. Additionally, a mechanistic understanding for why CTPS1 expression is elevated in advanced and resistant forms of many solid tumors is needed. Finally, the basis by which some cancer cells exhibit *de novo* or acquired resistance to STP938 remain to be elucidated.

## Conclusion

In conclusion, we conducted an unbiased and genome-wide approach through which we identified CTPS1 as an essential gene in many TNBC and ovarian cancer cell lines. These findings were validated using genetic and pharmacologic approaches whereby potent anti-neoplastic activity of the selective CTPS1 inhibitor, STP938, was demonstrated. Importantly, STP938 retained substantial activity at clinically relevant concentrations in physiologically relevant 3D culture systems and diminished tumor cell viability in *ex vivo* PDX models. Further, STP938 was shown to suppress tumor growth of an aggressive and therapy resistant PDX model *in vivo*. These results highlight a previously underexplored metabolic vulnerability and provide further rationale for the continued pre-clinical investigation and early-phase clinical evaluation of STP938 as both a monotherapy and combinatorial agent. Further translational studies aimed at identifying ideal biomarkers of sensitivity that can be employed for patient stratification, in addition to elucidating the mechanisms of *de novo* and acquired resistance, are warranted.

## Supplementary Material

Supplementary figures and tables.

## Figures and Tables

**Table 1:** Mean essentiality scores for indicated genes in TNBC and ERα^+^ cell lines contained within the DepMap Portal.

Gene	TNBC (Gene Effect)	ERα^+^ HER2^-^ (Gene Effect)	P-value
CTPS1	-0.949	-0.509	0.0023
HUS1	-0.734	-0.417	0.0251
PRKRA	-0.952	-0.553	0.0048
RAD1	-0.573	-0.323	0.0164
RAD9A	-1.254	-0.852	0.0095
RHOA	-0.526	-0.007	0.0044

**Table 2:** STP938 IC50s in a panel of breast and ovarian cancer cell lines.

BC Subtypes	BC Cell Lines	IC50 (nM)	OC Subtypes	OC Cell Lines	IC50 (nM)
TNBC	HS578T	111.9 ± 33.6	Serous	COV362	81.8 ± 30.4
TNBC	MDA-MB-231	142.0 ± 37.5	Serous	COV362 OlapR	91.4 ± 29.4
TNBC	MDA-MB-231-Par	132.3 ± 22.2	Serous	OVSAHO	138.3 ± 26.2
TNBC	MDA-MB-231-D-R	180.4 ± 29.4	Serous	OVSAHO#56 (Cis-R)	79.6 ± 16.9
TNBC	MDA-MB-231-P-R	330.2 ±128.6	Serous	PEO1	58.9 ± 10.4
TNBC	MDA-MB-436	74.2 ± 19.8	Serous	ABT^R^2 (pan-PARPi-R)	26.3 ± 5.5
TNBC	MDA-MB-468	130.7 ± 33.5	Endometrioid	MDAH2774	65.5 ± 11.6
TNBC	MDA-MB-468-Par	133.1 ± 73.7	Endometrioid	MDAH2774#42 (Cis-R)	128.1 ± 38.4
TNBC	MDA-MB-468-D-R	186.6 ± 4.6	Clear Cell	JHOC-5	362.1 ± 80.2
TNBC	MDA-MB-468-P-R	62.1 ± 25.42	Clear Cell	SKOV3	95.3 ± 9.8
TNBC	SUM159	98.7 ± 12.9	Mixed	IGROV1	552.1 ± 2.4
ERα^-^HER2^+^	SKBR3	826.9 ± 635.2	Mixed	IGROV1/CP (Cis-R)	81.9 ± 12.2
ERα^+^HER2^-^	MCF7	197.2 ± 45.68	Mixed	OV7	118.8 ± 22.3
ERα^+^HER2^-^	MCF7-AC1	601.2 ± 193.6			
ERα^+^HER2^-^	T47D	> 10 μM			
ERα^+^HER2^-^	SUM185	> 10 μM			
ERα^+^HER2^-^	ZR75-1	> 10 μM			

## Abbreviations

AIC: Akaike Information Criteria
BC: breast cancer
BIC: Bayesian Information Criteria
CRISPR: clustered regularly interspaced short palindromic repeats
CTP: cytidine triphosphate
CTPS1/CTPS2: cytidine triphosphate synthase 1/2
DFS: disease-free survival
ERα: estrogen receptor alpha
HGSOC: high-grade serous ovarian cancer
HER2: human epidermal growth factor receptor 2
HPRT1: hypoxanthine phosphoribosyltransferase 1
HUS1: checkpoint clamp component HUS1
IC50: half maximal inhibitory concentration
IACUC: Institutional Animal Care and Use Committee
LC-MS: liquid chromatography-mass spectrometry
OC: ovarian cancer
OS: overall survival
PARPi: poly (ADP-ribose) polymerase inhibitor
PDX: patient-derived xenograft
PRKRA: protein kinase: interferon-inducible double-stranded RNA dependent activator
PI: propidium iodide
qPCR: quantitative polymerase chain reaction
RAD1: cell cycle checkpoint protein RAD1
RAD9A: DNA repair protein RAD9A
REML: restricted maximum likelihood estimation
RHOA: Ras homolog family member A
RT-qPCR: reverse-transcription quantitative PCR
SSA: 5-sulfosalicylic acid
TCGA: The Cancer Genome Atlas
TNBC: triple-negative breast cancer
ULA: ultra-low attachment
UTP: uridine triphosphate

## References

[1] Arnold M, Morgan E, Rumgay H, Mafra A, Singh D, Laversanne M (2022). Current and future burden of breast cancer: Global statistics for 2020 and 2040. Breast.

[2] Sung H, Ferlay J, Siegel RL, Laversanne M, Soerjomataram I, Jemal A (2021). Global Cancer Statistics 2020: GLOBOCAN Estimates of Incidence and Mortality Worldwide for 36 Cancers in 185 Countries. CA Cancer J Clin.

[3] Lei S, Zheng R, Zhang S, Wang S, Chen R, Sun K (2021). Global patterns of breast cancer incidence and mortality: A population-based cancer registry data analysis from 2000 to 2020. Cancer Commun (Lond).

[4] Kim J, Harper A, McCormack V, Sung H, Houssami N, Morgan E (2025). Global patterns and trends in breast cancer incidence and mortality across 185 countries. Nat Med.

[5] Acheampong T, Kehm RD, Terry MB, Argov EL, Tehranifar P (2020). Incidence Trends of Breast Cancer Molecular Subtypes by Age and Race/Ethnicity in the US From 2010 to 2016. JAMA Netw Open.

[6] Yin L, Duan JJ, Bian XW, Yu SC (2020). Triple-negative breast cancer molecular subtyping and treatment progress. Breast Cancer Res.

[7] Howard FM, Olopade OI (2021). Epidemiology of Triple-Negative Breast Cancer: A Review. Cancer J.

[8] Furlanetto J, Loibl S (2020). Optimal Systemic Treatment for Early Triple-Negative Breast Cancer. Breast Care (Basel).

[9] Polley MC, Leon-Ferre RA, Leung S, Cheng A, Gao D, Sinnwell J (2021). A clinical calculator to predict disease outcomes in women with triple-negative breast cancer. Breast Cancer Res Treat.

[10] Obidiro O, Battogtokh G, Akala EO (2023). Triple Negative Breast Cancer Treatment Options and Limitations: Future Outlook. Pharmaceutics.

[11] Reddy SM, Barcenas CH, Sinha AK, Hsu L, Moulder SL, Tripathy D (2018). Long-term survival outcomes of triple-receptor negative breast cancer survivors who are disease free at 5 years and relationship with low hormone receptor positivity. Br J Cancer.

[12] Zagami P, Carey LA (2022). Triple negative breast cancer: Pitfalls and progress. NPJ Breast Cancer.

[13] Newman LA, Reis-Filho JS, Morrow M, Carey LA, King TA (2015). The 2014 Society of Surgical Oncology Susan G. Komen for the Cure Symposium: triple-negative breast cancer. Ann Surg Oncol.

[14] Dent R, Trudeau M, Pritchard KI, Hanna WM, Kahn HK, Sawka CA (2007). Triple-negative breast cancer: clinical features and patterns of recurrence. Clin Cancer Res.

[15] Stewart RL, Updike KL, Factor RE, Henry NL, Boucher KM, Bernard PS (2019). A Multigene Assay Determines Risk of Recurrence in Patients with Triple-Negative Breast Cancer. Cancer Res.

[16] Siegel RL, Kratzer TB, Wagle NS, Sung H, Jemal A (2026). Cancer statistics, 2026. CA Cancer J Clin.

[17] Caruso G, Weroha SJ, Cliby W (2025). Ovarian Cancer: A Review. JAMA.

[18] Palmqvist C, Staf C, Mateoiu C, Johansson M, Albertsson P, Dahm-Kahler P (2020). Increased disease-free and relative survival in advanced ovarian cancer after centralized primary treatment. Gynecol Oncol.

[19] Reid BM, Permuth JB, Sellers TA (2017). Epidemiology of ovarian cancer: a review. Cancer Biol Med.

[20] Lheureux S, Gourley C, Vergote I, Oza AM (2019). Epithelial ovarian cancer. Lancet.

[21] Fritz V, Fajas L (2010). Metabolism and proliferation share common regulatory pathways in cancer cells. Oncogene.

[22] Tsesmetzis N, Paulin CBJ, Rudd SG, Herold N (2018). Nucleobase and Nucleoside Analogues: Resistance and Re-Sensitisation at the Level of Pharmacokinetics, Pharmacodynamics and Metabolism. Cancers (Basel).

[23] Asnagli H, Minet N, Pfeiffer C, Hoeben E, Lane R, Laughton D (2023). CTP Synthase 1 Is a Novel Therapeutic Target in Lymphoma. Hemasphere.

[24] Martin E, Palmic N, Sanquer S, Lenoir C, Hauck F, Mongellaz C (2014). CTP synthase 1 deficiency in humans reveals its central role in lymphocyte proliferation. Nature.

[25] van Kuilenburg AB, Meinsma R, Vreken P, Waterham HR, van Gennip AH (2000). Isoforms of human CTP synthetase. Adv Exp Med Biol.

[26] van Kuilenburg AB, Meinsma R, Vreken P, Waterham HR, van Gennip AH (2000). Identification of a cDNA encoding an isoform of human CTP synthetase. Biochim Biophys Acta.

[27] Mullen NJ, Singh PK (2023). Nucleotide metabolism: a pan-cancer metabolic dependency. Nat Rev Cancer.

[28] Wu HL, Gong Y, Ji P, Xie YF, Jiang YZ, Liu GY (2022). Targeting nucleotide metabolism: a promising approach to enhance cancer immunotherapy. J Hematol Oncol.

[29] Lv L, Yang S, Zhu Y, Zhai X, Li S, Tao X (2022). Relationship between metabolic reprogramming and drug resistance in breast cancer. Front Oncol.

[30] Arafeh R, Shibue T, Dempster JM, Hahn WC, Vazquez F (2025). The present and future of the Cancer Dependency Map. Nat Rev Cancer.

[31] Posta M, Gyorffy B (2025). Pathway-level mutational signatures predict breast cancer outcomes and reveal therapeutic targets. Br J Pharmacol.

[33] Karthikeyan SK, Chandrashekar DS, Sahai S, Shrestha S, Aneja R, Singh R (2025). MammOnc-DB, an integrative breast cancer data analysis platform for target discovery. NPJ Breast Cancer.

[34] Anurag M, Jaehnig EJ, Krug K, Lei JT, Bergstrom EJ, Kim BJ (2022). Proteogenomic Markers of Chemotherapy Resistance and Response in Triple-Negative Breast Cancer. Cancer Discov.

[35] Etemadmoghadam D, deFazio A, Beroukhim R, Mermel C, George J, Getz G (2009). Integrated genome-wide DNA copy number and expression analysis identifies distinct mechanisms of primary chemoresistance in ovarian carcinomas. Clin Cancer Res.

[36] Cancer Genome Atlas Research N (2011). Integrated genomic analyses of ovarian carcinoma. Nature.

[37] Wang X, Emch MJ, Goetz MP, Hawse JR (2025). Anti-Neoplastic Activity of Estrogen Receptor Beta in Chemoresistant Triple-Negative Breast Cancer. Cancers (Basel).

[38] Rodman EPB, Emch MJ, Hou X, Bajaj A, Pearson NA, John AJ (2025). Lestaurtinib's antineoplastic activity converges on JAK/STAT signaling to inhibit treatment naive and therapy resistant forms ovarian cancer. NPJ Precis Oncol.

[39] Sonego M, Pellizzari I, Dall'Acqua A, Pivetta E, Lorenzon I, Benevol S (2017). Common biological phenotypes characterize the acquisition of platinum-resistance in epithelial ovarian cancer cells. Sci Rep.

[40] McGehee CD, Meng XW, Wu X, Correia C, Venkatachalam A, Flatten KS (2025). Codon specific readthrough as a mechanism of BRCA2 restoration in acquired PARP inhibitor and chemotherapy resistance. Nucleic Acids Res.

[41] Ray U, Thirusangu P, Jin L, Xiao Y, Pathoulas CL, Staub J (2023). PG545 sensitizes ovarian cancer cells to PARP inhibitors through modulation of RAD51-DEK interaction. Oncogene.

[42] O'Sullivan J, Kothari C, Caron MC, Gagne JP, Jin Z, Nonfoux L (2023). ZNF432 stimulates PARylation and inhibits DNA resection to balance PARPi sensitivity and resistance. Nucleic Acids Res.

[43] Weroha SJ, Becker MA, Enderica-Gonzalez S, Harrington SC, Oberg AL, Maurer MJ (2014). Tumorgrafts as in vivo surrogates for women with ovarian cancer. Clin Cancer Res.

[44] Venkatachalam A, Correia C, Peterson KL, Hou X, Schneider PA, Strathman AR (2024). Proapoptotic activity of JNK-sensitive BH3-only proteins underpins ovarian cancer response to replication checkpoint inhibitors. Mol Cancer.

[45] Hou X, Zanfagnin V, Xu C, Jessen E, Liu Y, Wang C (2025). Antitumor activity of rucaparib plus PLX038A in serous endometrial carcinoma. J Exp Clin Cancer Res.

[46] Oberg AL, Heinzen EP, Hou X, Al Hilli MM, Hurley RM, Wahner Hendrickson AE (2021). Statistical analysis of comparative tumor growth repeated measures experiments in the ovarian cancer patient derived xenograft (PDX) setting. Sci Rep.

[47] Utama RH, Atapattu L, O'Mahony AP, Fife CM, Baek J, Allard T (2020). A 3D Bioprinter Specifically Designed for the High-Throughput Production of Matrix-Embedded Multicellular Spheroids. iScience.

[48] Hurley RM, McGehee CD, Nesic K, Correia C, Weiskittel TM, Kelly RL (2021). Characterization of a RAD51C-silenced high-grade serous ovarian cancer model during development of PARP inhibitor resistance. NAR Cancer.

[49] AlHilli MM, Becker MA, Weroha SJ, Flatten KS, Hurley RM, Harrell MI (2016). In vivo anti-tumor activity of the PARP inhibitor niraparib in homologous recombination deficient and proficient ovarian carcinoma. Gynecol Oncol.

[50] Vogel C, Marcotte EM (2012). Insights into the regulation of protein abundance from proteomic and transcriptomic analyses. Nat Rev Genet.

[51] Shi DD, Savani MR, Abdullah KG, McBrayer SK (2023). Emerging roles of nucleotide metabolism in cancer. Trends Cancer.

[52] Robinson AD, Eich ML, Varambally S (2020). Dysregulation of de novo nucleotide biosynthetic pathway enzymes in cancer and targeting opportunities. Cancer Lett.

[53] Martin E, Minet N, Boschat AC, Sanquer S, Sobrino S, Lenoir C (2020). Impaired lymphocyte function and differentiation in CTPS1-deficient patients result from a hypomorphic homozygous mutation. JCI Insight.

[54] Sun Z, Zhang Z, Wang QQ, Liu JL (2022). Combined Inactivation of CTPS1 and ATR Is Synthetically Lethal to MYC-Overexpressing Cancer Cells. Cancer Res.

[55] Pfeiffer C, Grandits AM, Asnagli H, Schneller A, Huber J, Zojer N (2024). CTPS1 is a novel therapeutic target in multiple myeloma which synergizes with inhibition of CHEK1, ATR or WEE1. Leukemia.

[56] Lin Y, Zhang J, Li Y, Guo W, Chen L, Chen M (2022). CTPS1 promotes malignant progression of triple-negative breast cancer with transcriptional activation by YBX1. J Transl Med.

[57] Nakasuka F, Hirayama A, Makinoshima H, Yano S, Soga T, Tabata S (2024). The role of cytidine 5'-triphosphate synthetase 1 in metabolic rewiring during epithelial-to-mesenchymal transition in non-small-cell lung cancer. FEBS Open Bio.

[58] Nan Y, Wang K, Hu M, Luo Q, Wu X, Yu X (2026). Targeting de novo pyrimidine synthesis confers vulnerability to copper-mediated ATR inactivation in PARP inhibitor-resistant ovarian cancer. Nat Commun.

[59] Bigdeli M, Tremblay E, Provencher D, Mes-Masson AM, Rodier F (2026). Exploiting synthetic lethality in epithelial ovarian cancer: multi-dimensional approaches beyond DNA damage repair. Mol Cancer.

[60] Blaszczak E, Miziak P, Odrzywolski A, Baran M, Gumbarewicz E, Stepulak A (2025). Triple-Negative Breast Cancer Progression and Drug Resistance in the Context of Epithelial-Mesenchymal Transition. Cancers (Basel).

[61] Nedeljkovic M, Damjanovic A (2019). Mechanisms of Chemotherapy Resistance in Triple-Negative Breast Cancer-How We Can Rise to the Challenge. Cells.

[62] Kvokackova B, Remsik J, Jolly MK, Soucek K (2021). Phenotypic Heterogeneity of Triple-Negative Breast Cancer Mediated by Epithelial-Mesenchymal Plasticity. Cancers (Basel).

[63] Wu F, Mao Y, Ma T, Wang X, Wei H, Wang T (2022). CTPS1 inhibition suppresses proliferation and migration in colorectal cancer cells. Cell Cycle.

[64] Durand R, Bellanger C, Descamps G, Dousset C, Maiga S, Derrien J (2024). Combined inhibition of CTPS1 and ATR is a metabolic vulnerability in p53-deficient myeloma cells. Hemasphere.

[65] Liang JH, Ren YM, Du KX, Gao R, Duan ZW, Guo JR (2023). MYC-induced cytidine metabolism regulates survival and drug resistance via cGas-STING pathway in mantle cell lymphoma. Br J Haematol.

[66] Minet N, Boschat AC, Lane R, Laughton D, Beer P, Asnagli H (2023). Differential roles of CTP synthetases CTPS1 and CTPS2 in cell proliferation. Life Sci Alliance.

[67] Pickup MW, Mouw JK, Weaver VM (2014). The extracellular matrix modulates the hallmarks of cancer. EMBO Rep.

[68] Zhang M, Zhang B (2025). Extracellular matrix stiffness: mechanisms in tumor progression and therapeutic potential in cancer. Exp Hematol Oncol.

[69] Liang R, Song G (2023). Matrix stiffness-driven cancer progression and the targeted therapeutic strategy. Mechanobiol Med.

[70] Mathews CK (2006). DNA precursor metabolism and genomic stability. FASEB J.

[71] Bester AC, Roniger M, Oren YS, Im MM, Sarni D, Chaoat M (2011). Nucleotide deficiency promotes genomic instability in early stages of cancer development. Cell.

[72] Yague-Capilla M, Rudd SG (2024). Understanding the interplay between dNTP metabolism and genome stability in cancer. Dis Model Mech.

[73] Goel N, Foxall ME, Scalise CB, Wall JA, Arend RC (2021). Strategies in Overcoming Homologous Recombination Proficiency and PARP Inhibitor Resistance. Mol Cancer Ther.

[74] Yi T, Feng Y, Sundaram R, Tie Y, Zheng H, Qian Y (2019). Antitumor efficacy of PARP inhibitors in homologous recombination deficient carcinomas. Int J Cancer.

