# Supplementary material for: CTPS1 is an unexplored vulnerability in breast and ovarian cancer
Source: Theranostics. 2026 Jul 20;16(14):8195–214. doi: 10.7150/thno.134720 (PMC13440435; doi:10.7150/thno.134720)
Supplement: Supplementary file 1 [file thnov16p8195s1.pdf]

Table S1. siRNA Reagents Used for Gene Knockdown Experiments.

siRNA	Company	Catalog #	Lot #
siCTPS1	Dharmacon	L-006644-00-0005	200924
siHUS1	Dharmacon	L-003267-00-0005	200929
siNT	Dharmacon	D-001810-10-05	3253490
siPRKRA	Dharmacon	L-006426-00-0005	200924
siRAD1	Dharmacon	L-003293-00-0005	200924
siRAD9A	Dharmacon	L-003295-00-0005	200924
siRHOA	Dharmacon	L-003860-00-0005	200924

Table S2. Primer sequences for real-time quantitative PCR

Target Gene	Forward Primers (5'-3')	Reverse Primers (5'-3')
18S	AGCCTGAGAAACGGCTACCA	GGGTCGGGAGTGGGTAATTT
ACTB	CCCAGCCATGTACGTTGCTAT	TCACCGGAGTCCATCACGAT
CTPS1	TTGTTGGCCAAGATGTTGAA	TAGGTGTCCCTGGGTGAGAG
CTPS2	TGTCCCTCACATTACTGATGCT	GGGCTCTTCCTTATTACCATCCA
HUS1	CCTCCATTAGCCTCTGAAAGC	ACAGCGCAGGGATGAAATAC
PRKRA	AGCCAAAAGGAATGCTGCT	TGGTGGACAGTTCAGCAAGA
RAD1	GTTCCCACCTTGACTATCCCA	TTCTTCATCAGGGCAGCAGT
RAD9A	TCTGCCTATGCCTGCTTTCTCT	AGCGGAAGACAGACAGGAAAGAC
RHOA	CAGAAAAGTGGACCCCAGAA	CAAGACAAGGCACCCAGATT

Table S3. Patient-derived Xenograft (PDX) models minimal information standard (PDX-MI).

PDX Models (PH)	PH039	PH077	PH354	PH365	PH626	PH723
Gender	F	F	F	F	F	F
Age	62	70	60	73	59	51
Diagnosis	Fallopian Tube Carcinoma	Primary Peritoneal Carcinoma	Primary Peritoneal Carcinoma	Epithelial Carcinoma	Epithelial Carcinoma	Epithelial Carcinoma
Consent	Academic	Academic	Academic	Academic	Academic	Academic
Histology	Serous	Serous	Serous	Serous	Serous	Clear Cell/Undifferentiated
Grade	High	High	High	High	High	High
Stage	IIIC	IV	IIIC	IIIC	IIIC	IV
Treatment	Naïve	Naïve	Neo-adjuvant	Naïve	Naïve	Naïve

Mouse Strain	SCID-beige C.B.-17/ IcrHsd-*Prkdcscid Lystbg* ; ENVIGO	SCID-beige C.B.-17/ IcrHsd-*Prkdcscid Lystbg* ; ENVIGO	SCID-beige C.B.-17/ IcrHsd-*Prkdcscid Lystbg* ; ENVIGO	SCID-beige C.B.-17/ IcrHsd-*Prkdcscid Lystbg* ; ENVIGO	SCID-beige C.B.-17/ IcrHsd-*Prkdcscid Lystbg* ; ENVIGO	SCID-beige C.B.-17/ IcrHsd-*Prkdcscid Lystbg* ; ENVIGO
**Mouse Humanized**	No	No	No	No	No	No
**Preparation**	Solid Tumor	Solid Tumor	Solid Tumor	Solid Tumor	Solid Tumor	Solid Tumor
**Injection site**	IP	IP	IP	IP	IP	IP
**Characterization**	Histology	Histology	Histology	Histology	Histology	Histology
**Negative murine/EBV**	Yes	Yes	Yes	Yes	Yes	Yes

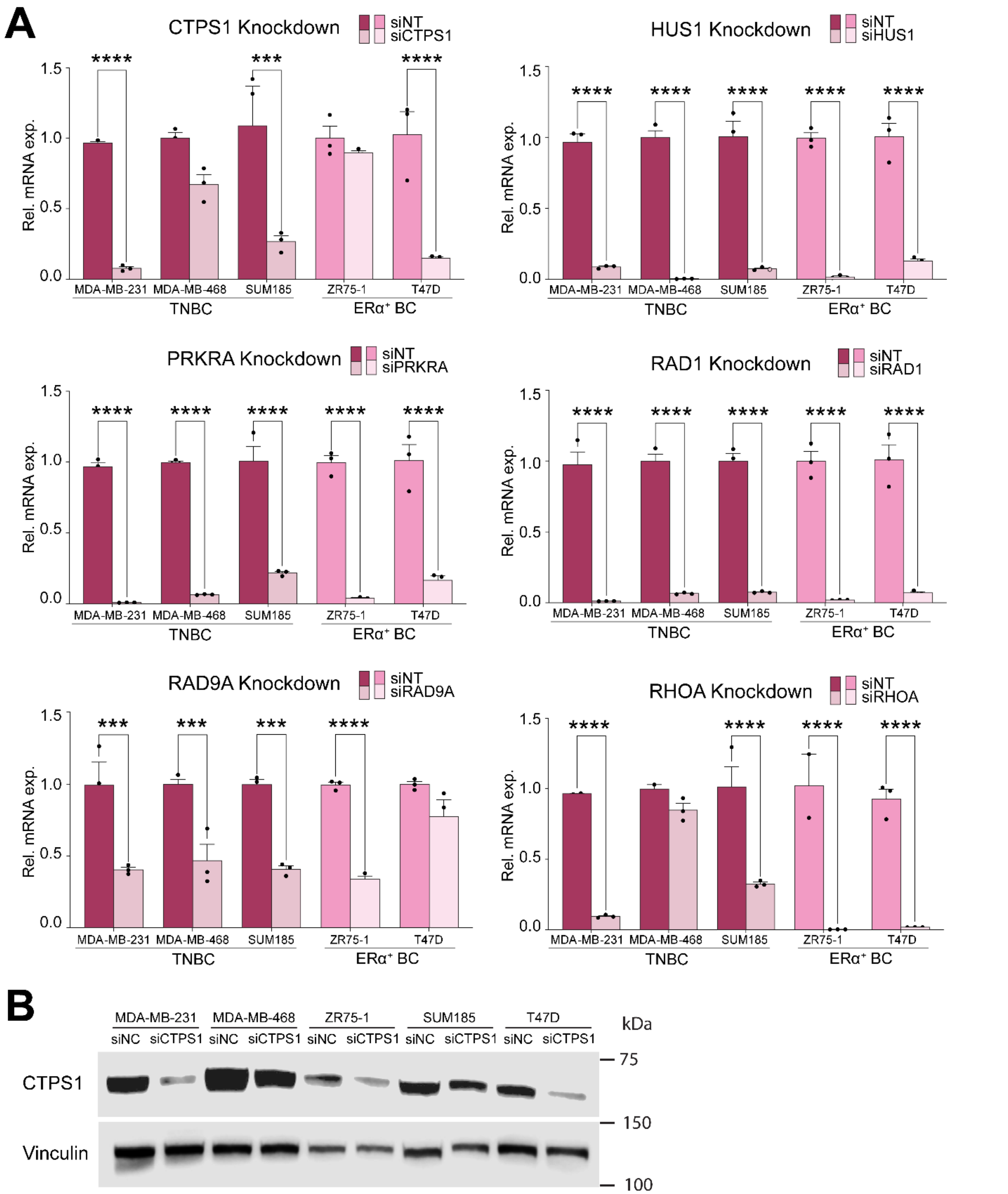

Figure S1. Efficiency of siRNA-mediated gene knockdown in breast cancer cell lines. (A) RT-qPCR analysis confirming suppression of CTPS1, HUS1, PRKRA, RAD1, RAD9A, and RHOA following transient siRNA

transfection in TNBC (MDA-MB-231, MDA-MB-468, SUM185) and ERα^{+}/HER2^{-} (ZR75-1, T47D) breast cancer cell lines. Cells were treated with 5 nM gene-specific SMARTpool siRNAs or a non-targeting control (siNT) for 72 h. Gene expression levels were normalized to 18S and ACTB and presented relative to siNT-treated controls for each cell line. Data represent mean ± SEM from three independent experiments. Statistical significance was determined using two-way ANOVA with correction for multiple comparisons. *** P<0.001, *** P<0.0001. (B) Representative western blot confirming CTPS1 knockdown following transient siRNA transfection in MDA-MB-231, MDA-MB-468, ZR75-1, SUM185, and T47D breast cancer cell lines. Vinculin was used as a loading control.

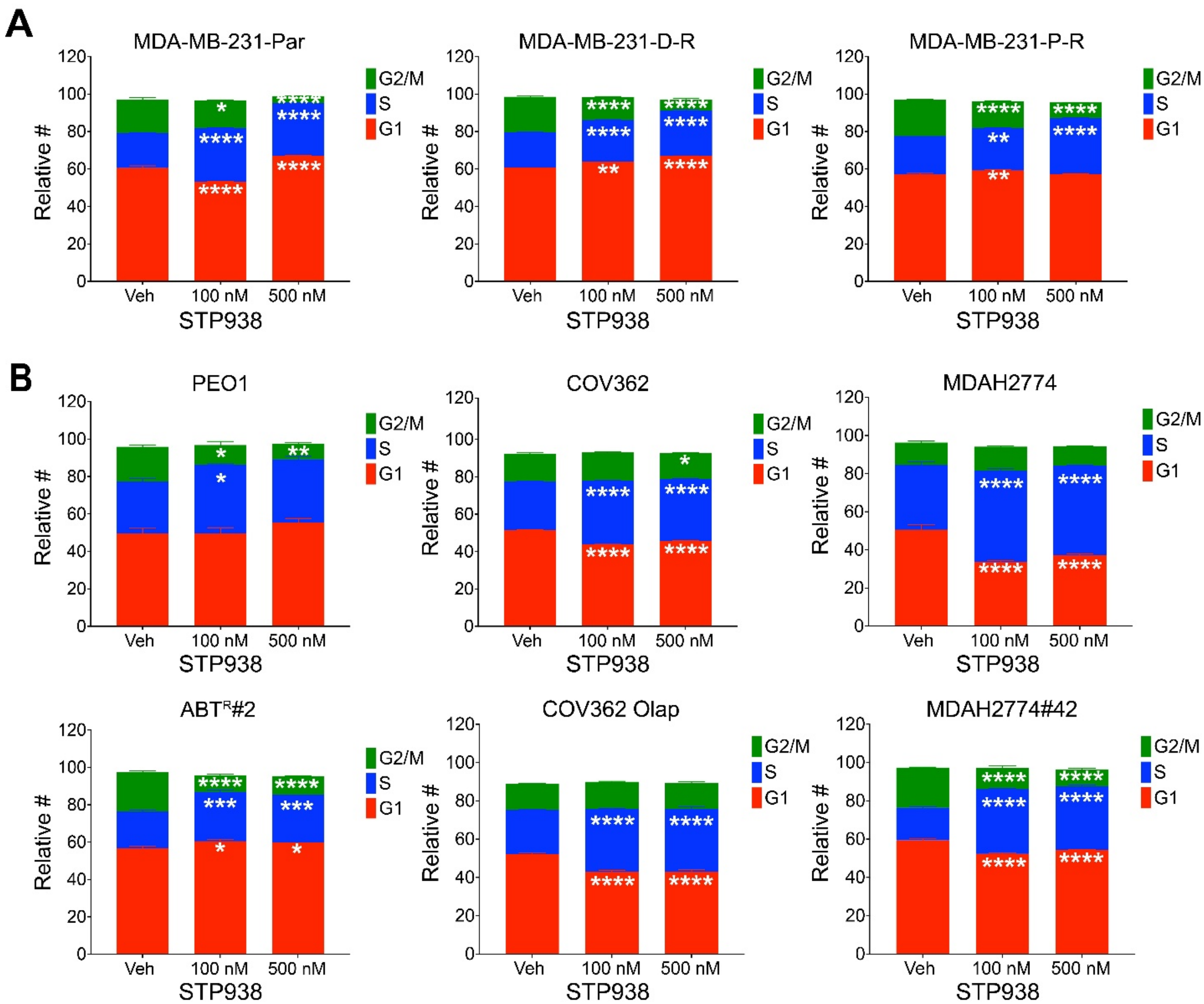

Figure S2. STP938 induces S-phase arrest in parental and therapy-resistant breast and ovarian cancer cell lines. Cell cycle analysis following 24 h treatment with vehicle (Veh), 100 nM, or 500 nM STP938 in parental and therapy-resistant models. (A) TNBC MDA-MB-231 parental (Par), doxorubicin-resistant (D-R), and paclitaxel-resistant (P-R) cells. (B) OC cell lines PEO1, COV362, MDAH2774, ABT^{R}#2 (cisplatin- and PARP inhibitor-resistant), COV362Olap (PARP inhibitor-resistant), and MDAH2774#42 (cisplatin-resistant). Cell cycle phase (G1, red; S, blue; G2/M, green) was quantified by propidium iodide staining followed by flow cytometry and expressed as relative percentage of total cells. Data are presented as mean ± SEM. Statistical significance was determined using two-way ANOVA with correction for multiple comparisons versus vehicle-treated controls. *$P < 0.05$; **$P < 0.01$; ***$P < 0.001$; ****$P < 0.0001$.
